# Overcoming barriers in glioblastoma: The potential of CAR T cell immunotherapy

**DOI:** 10.7150/thno.114257

**Published:** 2025-06-12

**Authors:** Muhammad Ijaz, Qingqin Tan, Yuqian Yan, Daoming Zhang, Qi Chen, Yinghe Zhang, Yanyang Tu, Bing Guo

**Affiliations:** 1 School of Science, Shenzhen Key Laboratory of Advanced Functional Carbon Materials Research and Comprehensive Application, Harbin Institute of Technology, Shenzhen-518055, China.; 2 Department of Blood Transfusion, Jiangxi Provincial People's Hospital, The First Affiliated Hospital of Nanchang Medical College, Nanchang, 330000, China.; 3 Jiangxi Provincial People's Hospital, The First Affiliated Hospital of Nanchang Medical College, Nanchang, 330000, China.; 4 Science Research Center, Huizhou Central People's Hospital, No. 41 E Ling North Road, Huizhou City, Guangdong Province, 516000 China.; 5 Science Research Center, Huizhou Central People's Hospital, Guangdong Medical University, Huizhou City, Guangdong Province, China.; 6 Huizhou Central People's Hospital Academy of Medical Sciences, Huizhou City, Guangdong Province, China.

**Keywords:** Glioblastoma, CAR T-cell therapy, Immunotherapy, Combination therapies, blood-brain barrier, Image-guided CAR-T therapy

## Abstract

Glioblastoma (GBM), the most aggressive and lethal primary brain tumor, is characterized by its high rate of growth, high genetic diversity, and resistance to conventional therapies. Chimeric antigen receptor (CAR) T cell immunotherapy has emerged as a promising treatment option for a variety of cancers, including GBM. However, CAR T therapy use in GBM is very challenging due to the unique challenges posed by the brain tumor microenvironment, including immune suppression, antigen heterogeneity, poor CAR T cell trafficking, and the blood-brain barrier (BBB). Advances in CAR T cell engineering, antigen screening, targeted administration, image-guided CAR-T therapy and combination therapies are transforming immunotherapy for GBM.AI-driven CAR T immunotherapy optimizes GBM treatment by enhancing target identification, therapy customization, and efficacy monitoring. This review aims to highlight the challenges hindering the success of CAR T cell therapy in glioblastoma and explore innovative strategies to enhance its efficacy, ultimately paving the way for more effective and durable treatment options for glioblastoma. We hope this review will stimulate interest among researchers and expedite the clinic translation of CAR T therapy of glioblastoma.

## Introduction

Despite intensive therapy glioblastoma (GBM), one of the deadliest types of primary brain tumors, has a median overall survival (mOS) of around 15 months 1. Therapeutic effectiveness is severely hampered by the tumor's highly infiltrative nature, wide heterogeneity, and inherent resistance to standard treatments including radiation, temozolomide-based chemotherapy, and surgical excision. There is an urgent need for novel and more efficient therapeutic approaches as a result of this dire outlook 2,3.

Immunotherapy has been a ground-breaking method for treating cancer throughout the last ten years. Immunotherapy represents a transformative approach in modern medicine, leveraging the body's own immune system to recognize and eliminate cancer cells with precision and durability 4. CAR-T cell treatment has attracted a lot of interest due to its exceptional efficacy in treating multi hematological cancers including B-cell acute lymphoblastic leukemia, diffuse large B-cell lymphoma, and multiple myeloma 5. In order to guide a powerful and targeted immune response, CAR T cells are genetically modified to produce synthetic receptors that identify and bind certain antigens on tumor cells 6. With impressive results in hematological malignancies, CAR T cell treatment has become a game-changing strategy in cancer. Because CAR T cells are designed to specifically target antigens found in tumors, they can effectively and precisely destroy cancer cells through immune-mediated means. They are a desirable treatment alternative due to their versatility and potential for sustained anticancer action 7, 8. Nevertheless, there have been several difficulties in using CAR T cells to treat solid tumors like GBM 9-11. Because of particular obstacles such as antigen heterogeneity, the immunosuppressive tumor microenvironment (TME) successful application to solid tumors, including GBM, has proven more difficult 12. Checkpoint inhibitors have revolutionized cancer immunotherapy by targeting regulatory pathways like PD-1/PD-L1 and CTLA-4 to restore T cell function. While they show remarkable success in various cancers, their efficacy in glioblastoma remains limited due to the tumor's immunosuppressive environment and poor immune cell infiltration into the central nervous system 13.

In addition, the protective BBB, which restricts the administration of systemic medicines and protects the tumor from the host immune response, is another particular difficulty associated with GBM's) 14. Additionally, other cancer treatments, like those based on proteins and antibodies like doripenem are unsuccessful for GBM also because they are unable to cross the BBB 15. Hence, CAR-T cells were restricted in the ability to penetrate the brain, and limit the therapeutic efficiency of CAR-T therapy in GBM. The extensive infiltration of CAR T cells into adjacent healthy brain tissue complicates the precise targeting and distribution of these cells to glioblastoma (GBM) cells, increasing the risk of toxicity and off-target effects. The extensive infiltration of CAR T cells into adjacent healthy brain tissue complicates the precise targeting and distribution of these cells to glioblastoma (GBM) cells, increasing the risk of toxicity and off-target effects 16. Despite these challenges, new developments in genetic engineering, combinatorial treatment approaches, and creative delivery methods are raising hope for using CAR T cells to treat GBM 17. The immunosuppressive milieu of glioblastoma (GBM) hinders effective anti-tumor immune responses. It is characterized by regulatory T cells, myeloid-derived suppressor cells, and inhibitory cytokines that suppress T cell activity. Additionally, GBM-induced expression of immune checkpoints creates a hostile environment, limiting the efficacy of immunotherapies such as CAR T-cell therapy 18.

One tactic to get over these obstacles is image-guided CAR-T treatment. This method uses cutting-edge imaging techniques to track CAR-T cells in real time, including positron emission tomography (PET), magnetic resonance imaging (MRI), and fluorescence imaging. With the use of these technologies, CAR-T cells can be precisely delivered to tumor areas, their biodistribution can be better monitored, and therapy can be modified as needed 19. Additionally, by making it easier to identify remaining tumor sites, image guiding increases therapy specificity while reducing off-target effects. Image-guided CAR-T therapy may improve treatment efficacy and safety, according to preclinical and early clinical research. In addition to addressing delivery and monitoring issues, combining real-time imaging with CAR-T therapy opens the door to individualized treatment plans catered to each patient's particular GBM dynamics 20.

Furthermore, AI-driven CAR T immunotherapy offers a promising approach to glioblastoma treatment by enhancing therapy design and monitoring. AI algorithms enable precise identification of tumor-specific antigens, optimizing CAR T cell engineering for improved targeting and reduced off-target effects. Additionally, AI aids in personalizing treatment strategies, predicting patient responses, and analyzing real-time data to monitor therapy efficacy and manage side effects, ultimately improving clinical outcomes in this aggressive cancer 21.

**Scheme 1:**
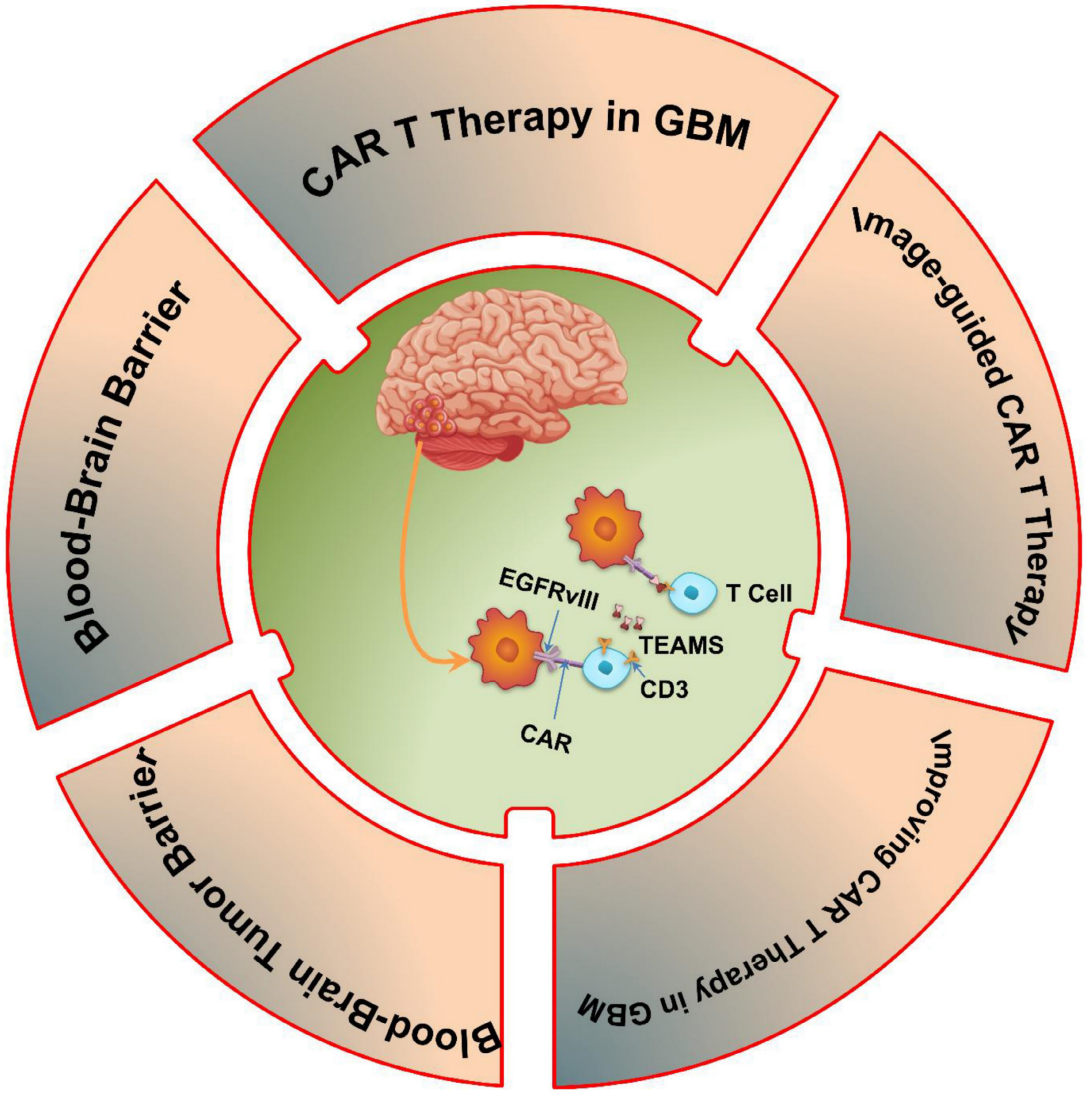
This schematic illustrates CAR T-cell immunotherapy for GBM, highlighting TEAMs (T-cell Engaging Molecules) used as adapters, such as anti-EGFR and anti-CD3.

Therefore, the purpose of this review is to investigate the possibility of CAR T cell immunotherapy as a game-changing method for treating GBM. We will point out significant developments in CAR T cell engineering, and assess new approaches meant to get over these obstacles. This study aims to shed light on how CAR T cell immunotherapy might change the treatment landscape for GBM and provide patients fighting this difficult illness fresh hope by analyzing the field's limits, prospects, and future directions **(Scheme 1)**.

## CAR T Therapy

**Figure 1:**
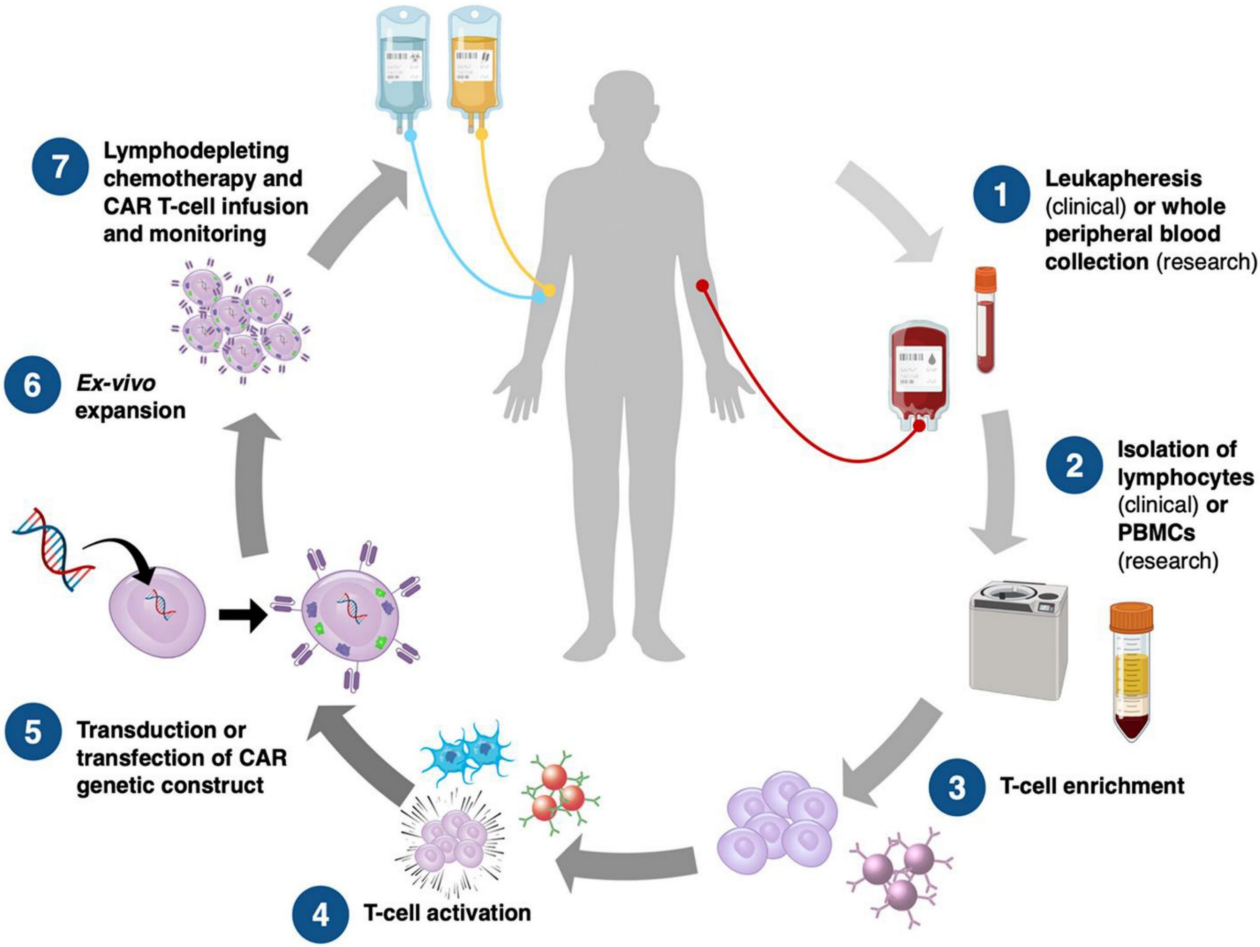
The process of CAR T-cell manufacture, from sample collection to patient reinfusion. PBMCs, peripheral blood mononuclear cells. Adapted with permission from reference 27. Copyrights 2023, WILEY.

Chimeric antigen receptors (CARs) are engineered synthetic receptors that enable T lymphocytes to recognize and eliminate cells expressing specific antigens, bypassing the need for antigen presentation via major histocompatibility complex (MHC) molecules 22. A revolutionary development in immunotherapy, CAR T cell treatment is designed to improve the immune system's capacity to identify and eliminate cancer cells. In order to precisely target tumor-specific antigens regardless of major histocompatibility complex (MHC) presentation, this approach is based on genetically altering a patient's T cells to produce synthetic receptors, or CARs 23, 24. The initial generation of CAR T cells had a straightforward design, consisting of an intracellular signaling region, usually CD3, and a transmembrane domain fused to an extracellular antigen-binding domain generated from a single-chain variable fragment (scFv) of an antibody 25. However, since they were unable to maintain T cell activation and proliferation, these early designs had limited effectiveness** (Figure 1).** Later developments resulted in the creation of second- and third-generation CARs, which greatly increased T cell survival, persistence, and antitumor activity by integrating co-stimulatory domains like CD28 and 4-1BB (CD137) 26.

**Figure 2:**
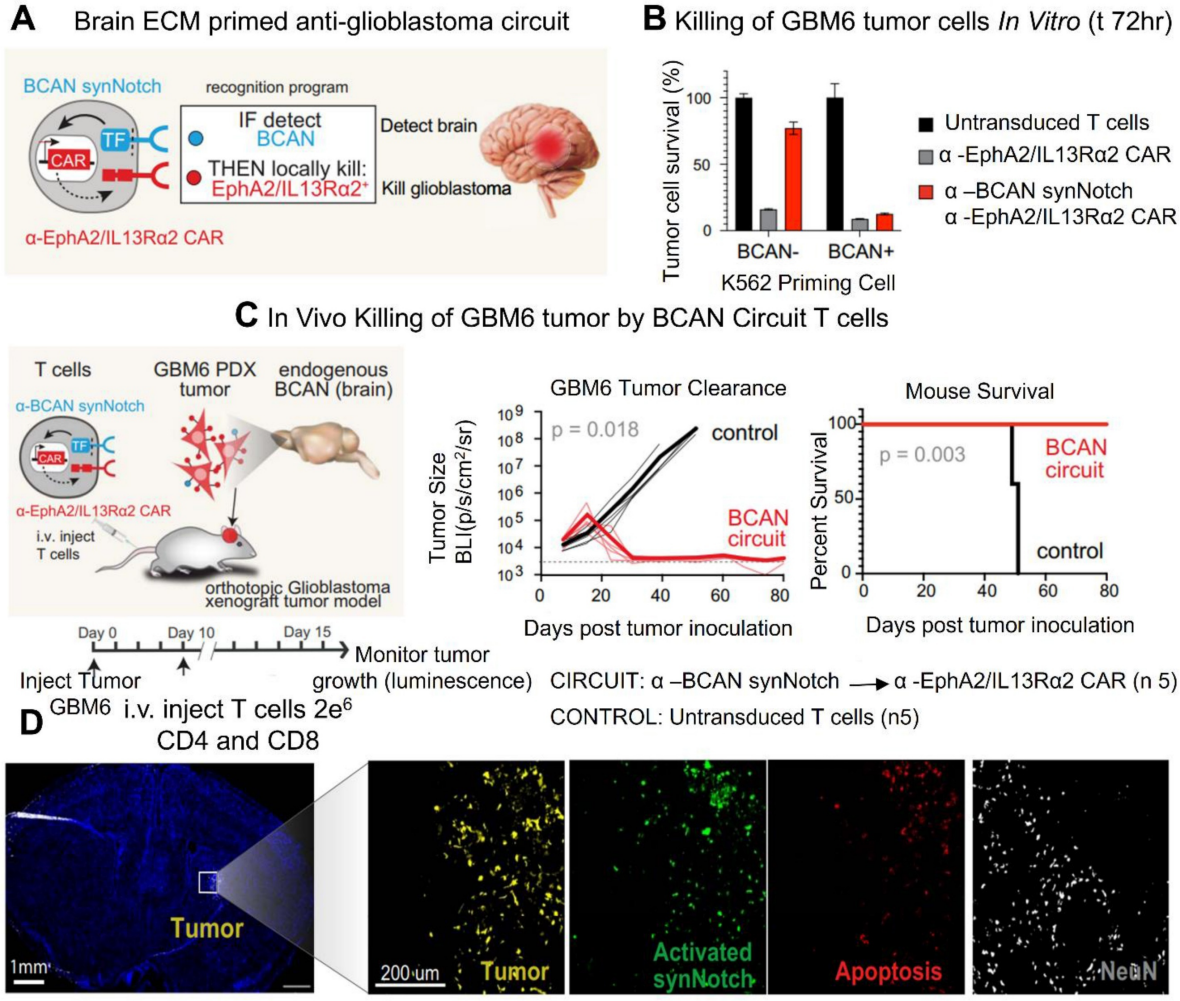
CAR anticancer action is directed precisely and potently to the intracerebral GBM PDX by SynNotch identification of the CNS-specific ECM molecule BCAN. **(A)** A CAR T cell that targets the brain. Only in the brain was anticancer CAR expression induced by the a-BCAN synNotch receptor. **(B)** *In vitro* destruction of GBM6 PDX tumors. GBM6 target cells and K562 priming cells, either expressing or not expressing BCAN, were cocultured with primary CD8^+^ T cells transduced with the a-BCAN synNotch→a-EphA2/IL13Ra2 CAR circuit (or with the constitutively expressed a-EphA2/IL13Ra2 CAR). **(C)** GBM6 tumor elimination *in vivo*. NCG mice's brains were orthotopically implanted with GBM6 tumors that expressed luciferase and mCherry. **(D)** Ten days following a-BCAN SynNotch→CAR T cell infusion (3 million CD4^+^ and CD8^+^ cells each), GBM6 tumor-bearing animals were put to death. Reused under Creative Commons Attribution License (CC BY) 28.

CAR T cell therapy has emerged as a promising strategy for treating a range of diseases. T cells possess the unique ability to infiltrate tissues, sense external signals, and modulate the tissue microenvironment. By engineering T lymphocytes to recognize specific antigens and locally release therapeutic agents, treatment efficacy can be enhanced while minimizing systemic off-target effects. This approach leverages molecular markers to direct T cells in delivering precise therapies. Synthetic Notch (synNotch) receptors have been designed to detect particular signals and initiate a customized transcriptional response. In preclinical models, these engineered T cells have demonstrated therapeutic potential. This cell-based strategy offers dual specificity molecular targeting through the therapeutic payload and spatially restricted activation. By mimicking the body's natural biological selectivity, this approach improves targeting accuracy within affected tissues and reduces the risk of systemic side effects** (Figure 2)** 28.

## CAR T Therapy in GBM

CAR-T therapy has revolutionized cancer treatment by harnessing the immune system to selectively target tumor cells 29. This method has demonstrated exceptional efficacy in treating hematological malignancies, resulting in FDA-approved therapies for lymphomas and leukemias 30, 31. Glioblastoma (GBM) is the most aggressive and deadly primary brain tumor. It is characterized by significant heterogeneity, rapid growth, and resistance to standard treatment modalities 32. Despite advancements in surgical resection, chemotherapy, and radiation therapy, patients with GBM continue to have a poor prognosis, with a median overall survival of only 12 to 15 months 33. The persistent challenge in treating this disease underscores the critical need for the development of more effective therapeutic strategies 34. By reprogramming T cells to identify and target GBM cells, CAR-T treatment offers a potential immunotherapeutic approach to overcome these obstacles 35. One of the main therapeutic challenges for GBM is the highly immunosuppressive TME, which restricts the effectiveness of traditional immune-based therapy. To avoid immune monitoring, GBM cells use a variety of strategies, including the release of immunosuppressive cytokines and the overexpression of immunological checkpoint molecules like Programmed death-ligand 1 (PD-L1), Cytotoxic T-lymphocyte-associated protein 4 (CTLA-4) and T-cell immunoglobulin and mucin-domain containing-3 (TIM-3) 36, 37. By modifying T cells to express receptors that selectively target tumor-associated antigens (TAAs) such Epidermal Growth Factor Receptor Variant III (EGFRvIII), Interleukin-13 Receptor Alpha 2 (IL-13Rα2) and Human Epidermal Growth Factor Receptor 2 (HER2), CAR-T therapy seeks to overcome these obstacles 38. Although preclinical and early clinical trials have shown encouraging anti-tumor effectiveness, antigen heterogeneity and adaptive resistance mechanisms have restricted the duration of CAR-T responses in GBM patients 39.

**Figure 3:**
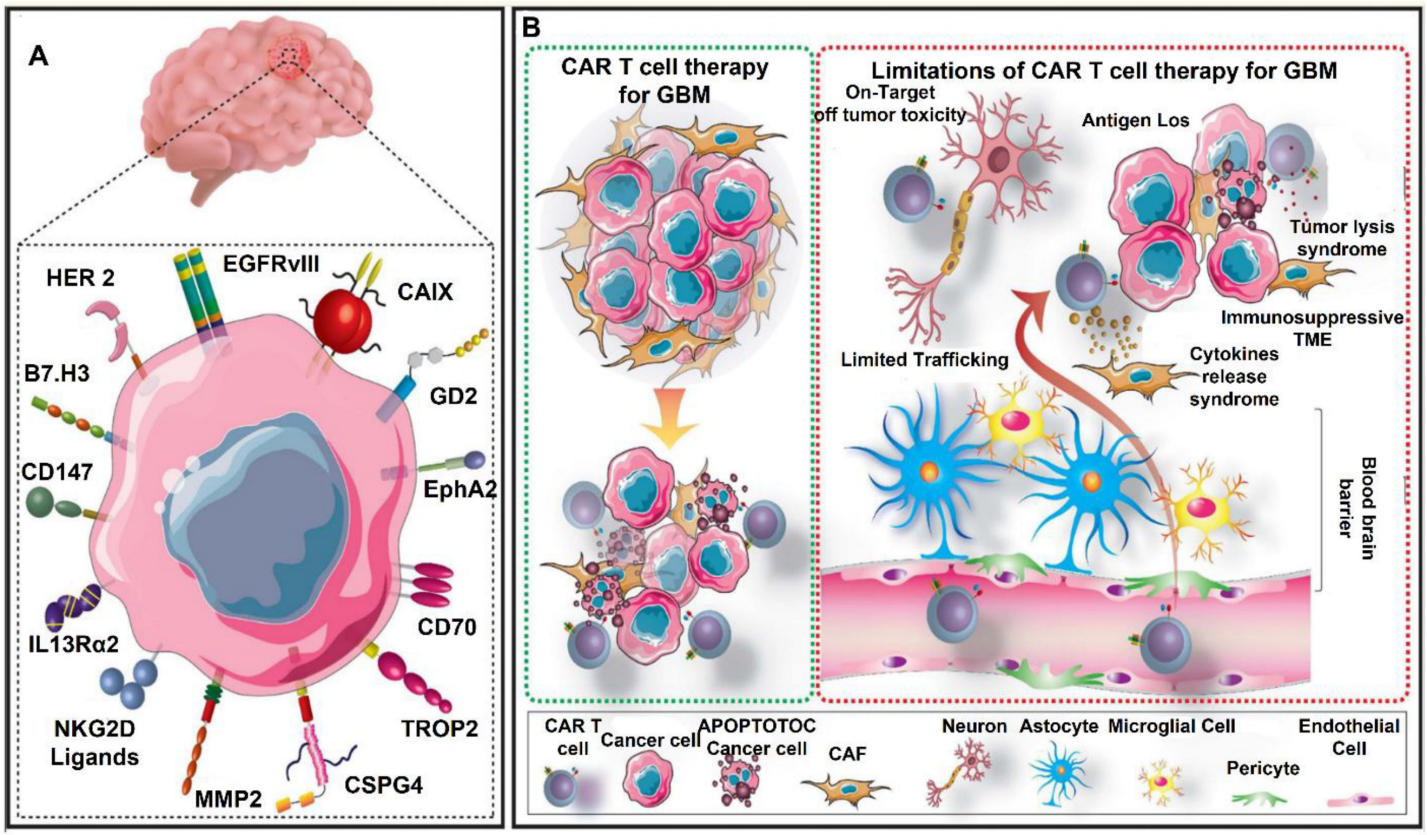
An overview of GBM therapy with CAR T cells. **(A)** Numerous targetable TAs expressed by GBM cells have been evaluated in preclinical or clinical CAR T cell investigations Patients can receive TA-specific CAR T cells to identify and eradicate GBM cells that express TA **(B)**. However, this technique is hampered by a number of restrictions. These challenges include tumor lysis syndrome, cytokine release syndrome, on-target/off-tumor toxicity, limited penetration across the blood-brain barrier, an immunosuppressive tumor microenvironment (TME), and the loss of target antigens. Reused under Creative Commons Attribution License (CC BY) 46.

The limited trafficking and penetration of CAR-T cells into the brain as a result of the BBB and the thick extracellular matrix of GBM is another major barrier 40. To improve T-cell penetration and persistence within the tumor, techniques including intracranial or intraventricular CAR-T cell administration have been investigated 41, 42. Furthermore, new developments in gene-editing methods like CRISPR CaS-9 technology, cytokine such as IL-7 and IL-15 support for CAR-T cells, and checkpoint inhibitor such as PD-1 or CTLA-4 combo therapy have demonstrated promise in enhancing their effectiveness 43, 44. To maximize CAR-T therapy for the treatment of GBM, issues such on-target/off-tumor toxicity, T-cell fatigue, Cytokine release syndrome (CRS) and immune-related side effects must be resolved despite these advancements 45. In conclusion, CAR-T cell therapy is a potential yet challenging treatment option for GBM. Although it provides a focused strategy for getting around the drawbacks of traditional treatments, a number of biological and technological obstacles need to be overcome in order to achieve long-term therapeutic success. The advancement of CAR-T treatment for GBM patients will depend heavily on ongoing research initiatives aimed at improving T-cell persistence, optimizing CAR designs, and modifying the immunosuppressive TME **(Figure 3)**.

According to Montoya *et al*. (2024), early research focused on IL13Rα2 since it was common in GBMs and was linked to aggressive tumor behavior and a bad prognosis. In 2015, the first pilot trial evaluating safety and feasibility examined the effectiveness of CD8^+^ CAR-T-cells targeting IL13Rα2 in treating three patients with recurrent GBM. The therapy's safety profile was good when CAR-T cells were administered intracranially into the resection cavity. Although two patients saw a temporary decrease in GMB activity, analysis of tumor tissue from one patient revealed a drop in total IL13Rα2 expression 47. All treated individuals had a GBM recurrence in spite of these positive results. In another study, CAR-T-cells that target IL13Rα2 were administered many times to a patient with recurrent multifocal GBM using two different intracranial delivery methods. This included infusions first into the cavity of the removed tumor and then into the ventricular system. Notably, the CAR-T-cell treatment was not associated with any toxicities of Grade 3 or above. A positive clinical response followed, with all cerebral and spinal tumors regressing, and this response lasted for 7.5 months after the start of CAR-T-cell treatment **Table 1** 48.

**Figure 4:**
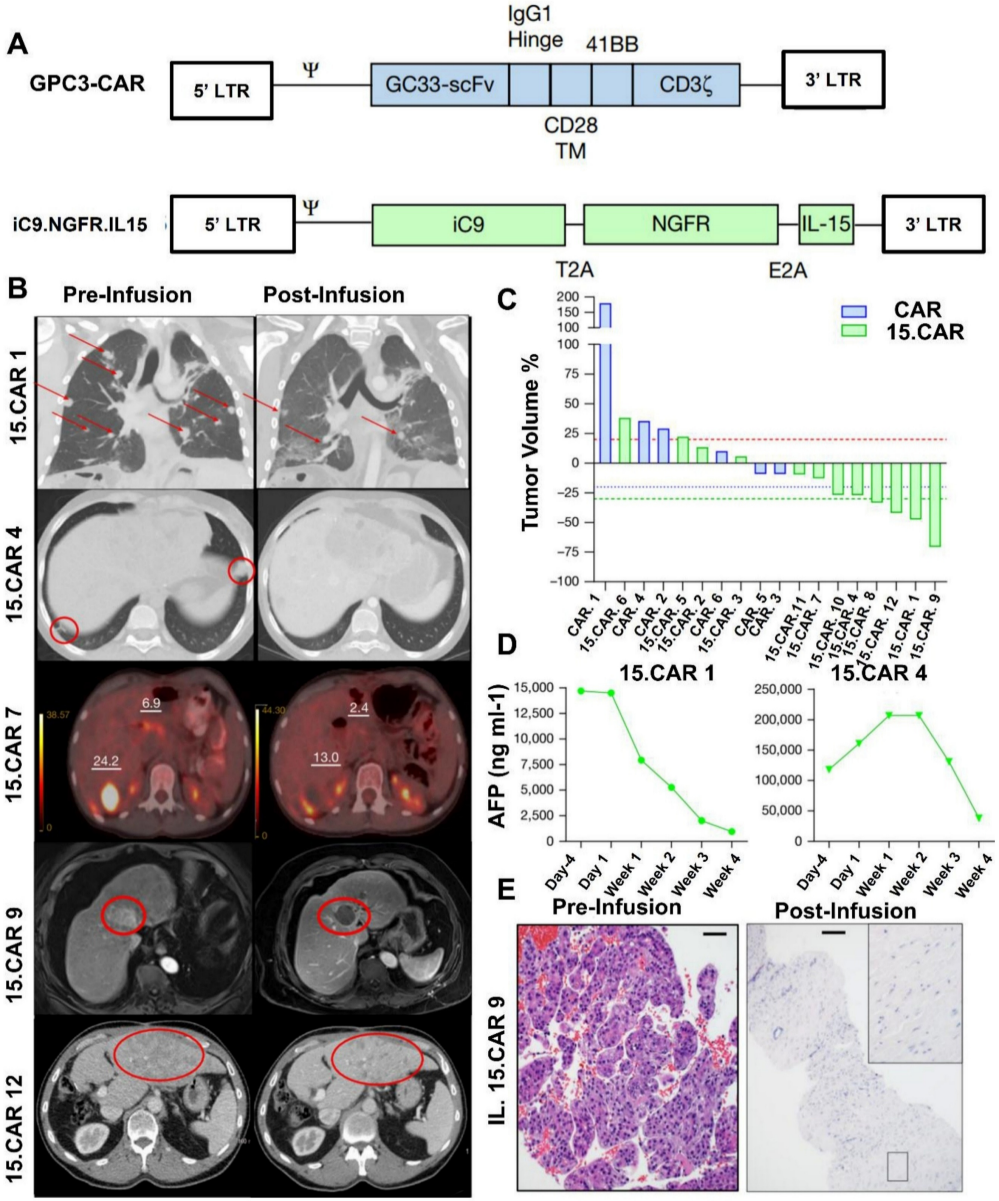
**(A)** GPC3 CAR and iC9.NGFR.IL-15 transgene maps are utilized to co-transduce T cells and produce infusion products. Transmembrane domain (TM). By comparing three-dimensional imaging before and after infusion, antitumor responses were ascertained. **(B)**, MRI abdomen (15.CAR9), axial CT abdomen (15.CAR12), PET-CT (15.CAR7), axial CT chest (no. 15.CAR4), and coronal computed tomography (CT) images of the chest (15.CAR1) demonstrating before and after CAR T-cell infusion. Tumors are shown by red circles and arrows. The standardized uptake levels of liver tumors displayed by PET are represented by numbers. **(C)**. A waterfall plot showing the variations in tumor volumes of patients receiving treatment with either 15.CAR T cells or 3 × 107 m-2 CAR. 20% rise is represented by a red dashed line, 20% reduction by a blue dashed line, and 30% drop by a green dashed line. **(D)**, Serum AFP levels in responders with AFP-secreting tumors at the specified intervals. **(E)** Haematoxylin and eosin staining was used to evaluate the pre- (left) and post-infusion tumor biopsies (right), which was carried out once in the clinical pathology laboratory. The results indicated that the liver tumor of patient number 15.CAR-9 had nearly total necrosis. 500 µm scale bars before infusion and 1,000 µm scale bars after infusion. Reused under Creative Commons Attribution License (CC-BY) 56.

According to a study by Steffin *et al*. (2024), in preclinical models of solid neoplasms where CAR T cells have low efficiency, interleukin-15 (IL-15) increases the antitumor characteristics of CAR T cells and encourages the survival of T lymphocytes. They evaluate the impact of IL-15 co-expression on Glypican-3 (GPC3)-targeted CAR T cells here after referred to as GPC3 CAR T cells in human subjects. GPC3 is found in a class of solid tumors. GPC3 CAR T cells were administered to Cohort 1 patients (NCT02905188 and NCT02932956); these cells were safe, although they did not exhibit any objective antitumor response, and they peaked in growth after two weeks. GPC3 CAR T cells that co-expressed IL-15 (15.CAR) were given to Cohort 2 patients (NCT05103631 and NCT04377932). This resulted in a 33% antitumor response rate and a 66% disease control rate, driven by considerably enhanced cell multiplication. CAR T-cell infusion was linked to a higher incidence of cytokine release syndrome (CRS). Respondents' CAR T cells displayed overexpression of JUN and FOS family members, as well as genes linked to type I interferon signaling, and inhibition of SWI/SNF epigenetic regulators **(Figure 4)**. All of these findings show that IL-15 promotes GPC3 CAR T-cell growth, intratumor survival, and antitumor efficacy in patients 56.

## The Blood-Brain Barrier Blocks CAR-T Cells

The CNS is shield by the BBB, a highly specialized and selective mechanism that preserves homeostasis while blocking the entry of dangerous chemicals 57. The BBB, which is made up of pericytes, astrocyte end-feet, and endothelial cells connected by tight junctions, makes it difficult to deliver therapeutic medicines like CAR T cells to brain malignancies like GBM 58. The tight junctions and efflux transporters in the BBB severely restrict the capacity of CAR T cells to penetrate the barrier and reach tumor locations when they are given intravenously. Only a tiny percentage of these modified immune cells are able to move to the CNS, according to studies, which frequently leads to sub-therapeutic concentrations at the tumor site 59. Additionally, while GBM's aggressive nature causes some BBB disruption, this disruption is uncertain and inadequate for consistent CAR T cell distribution, leaving certain tumor locations poorly targeted 60.

Several strategies have been developed to improve the delivery of CAR T cells to the central nervous system (CNS) in an effort to overcome these challenges 61. One method is to inject CAR T cells directly into the tumor site or cerebrospinal fluid (CSF), completely circumventing the BBB. For example, intracranial or intraventricular delivery strategies have been used in clinical studies targeting IL-13 receptor alpha 2 (IL-13Rα2) and HER2 in GBM patients, which has led to increased CAR T cell accumulation at the tumor site and, in certain cases, tumor regression 62. And, the intraventricular administration of HER2-targeted CAR T cells was in a phase I study for CNS malignancies, including GBM. Engineering CAR T cells to express chemicals that promote BBB traversal is another interesting tactic. By taking advantage of the chemokine gradients generated by GBM cells, CAR T cells that have been altered to express the chemokine receptor CXCR4 have demonstrated enhanced trafficking to CNS malignancies 63.

**Figure 5:**
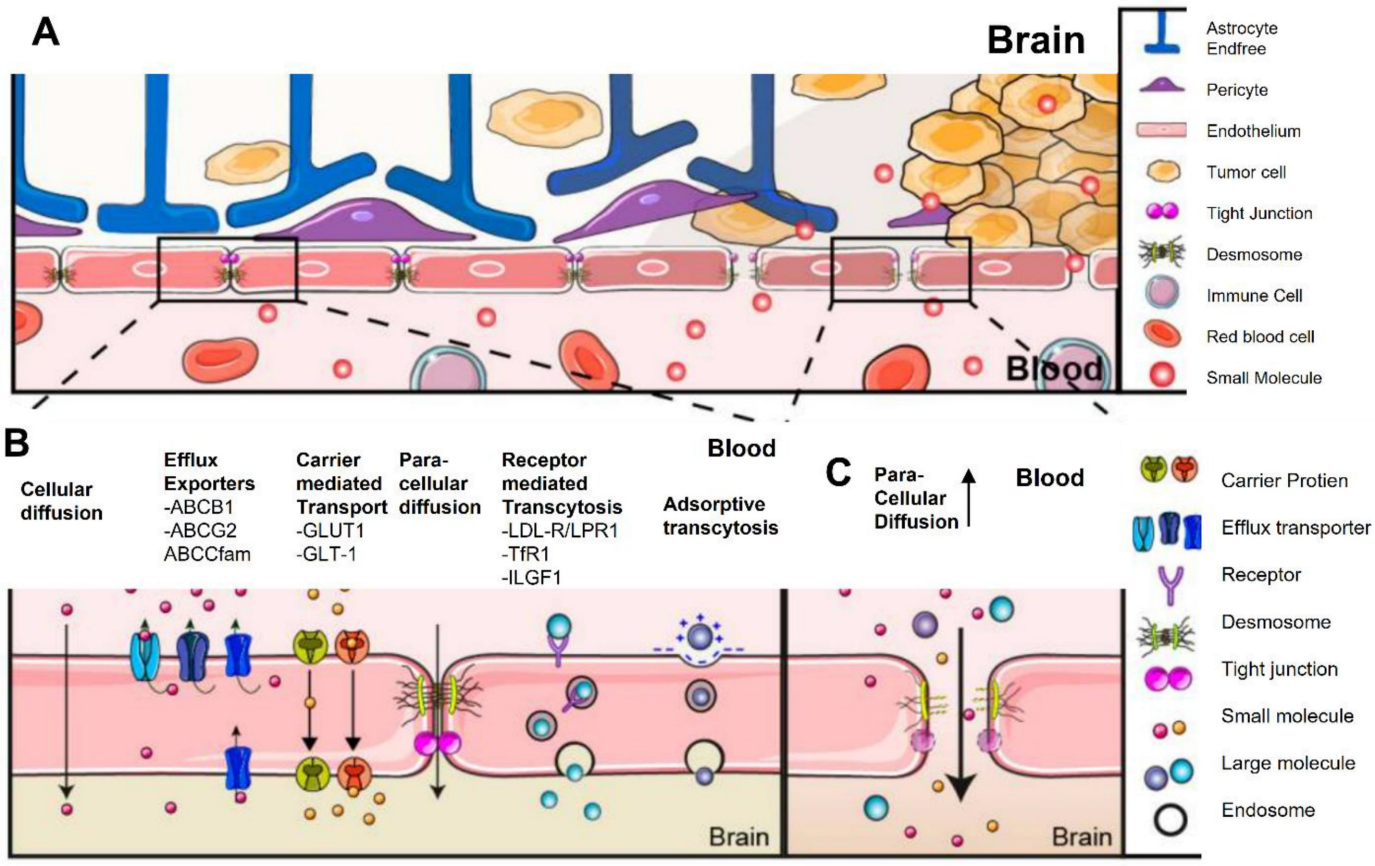
The brain barrier of blood tumors and the blood-brain barrier. Diagrammatic representation of the blood-tumor and BBB. **(A)** The neurovascular unit, which is made up of endothelial cells, pericytes, and astrocytes that communicate with neurons, maintains an unbroken BBB under normal circumstances **(B)** Under normal circumstances, the blood-brain barrier is made up of intact endothelial cells that are joined by adherence and tight junctions, which inhibits the majority of paracellular transport. **(C)** The blood-brain tumor barrier with enhanced paracellular transport as a result of tight junction loss and neurovascular unit disarray. Reused under Creative Commons Attribution License (CC BY) 74.

Novel approaches to temporarily interfere with the BBB have also been investigated. The BBB is momentarily opened by methods like focused ultrasound (FUS) in conjunction with microbubbles, which improves the penetration of CAR T cells 64. FUS enhances CAR T cell infiltration into CNS malignancies, improving tumor control, according to preclinical research in mouse models of GBM 65. For example, intravenous administration of EGFRvIII-targeted CAR T cells in studies showed low efficiency because of inadequate BBB penetration, highlighting the necessity of intratumoral injections as an alternate delivery route 66. Nevertheless, preclinical research employing focused ultrasound showed enhanced delivery of CAR T cells and better treatment results in models of CNS tumors** (Figure 5)**. Furthermore, scientists have created "armored" CAR T cells that release immune checkpoint inhibitors or cytokines like IL-15. In addition to altering the TME, these cells may also raise BBB permeability, which would allow more immune cells to infiltrate 67-70. Although the BBB continues to be a major barrier to the use of CAR T treatment for GBM, new developments in molecular engineering, creative delivery strategies, and approaches for localized BBB regulation provide encouraging avenues for progress 71. There is promise for overcoming this significant obstacle and realizing the full potential of immunotherapy for CNS malignancies by combining these strategies with CAR T cell technology 72, 73.

## CAR T Therapy Evasion in GBM

The success of CAR T-cell therapy in treating hematological malignancies has driven both preclinical and clinical research efforts to explore its potential in glioblastoma (GBM). However, there hasn't been any significant success in recent experiments. Because GBM may avoid immune responses in a number of ways, it presents serious obstacles to the effectiveness of CAR T-cell treatment 75, 76. Antigen escape is a crucial tactic, in which GBM cells suppress or cease to express the target antigen. CAR T-cells that target the common GBM-specific antigen (EGFRvIII), for instance, frequently encounter resistance as tumor cells adapt by changing or decreasing EGFRvIII expression, which results in therapeutic failure. In order to reduce antigen escape, researchers are investigating multi-targeting strategies that combine CAR T-cells guided by EGFRvIII with those that target additional antigens, such as IL-13Rα2 or HER2 77.

A further significant obstacle is the TME, which suppresses CAR T-cell function via a variety of mechanisms, including myeloid-derived suppressor cells (MDSCs), regulatory T-cells (Tregs), and immunosuppressive cytokines including VEGF, IL-10, and TGF-β. These components inhibit the cytotoxicity, proliferation, and activation of CAR T cells 78. To further inhibit immune responses, TGF-β generated within the TME, for example, recruits Tregs and reduces the efficacy of CAR T-cells. Furthermore, GBM cells commonly express PD-L1, which binds to the CAR T-cell's PD-1 receptor and results in fatigue. Combining CAR T-cells with immune checkpoint inhibitors (such as anti-PD-1 antibodies) or genetically modifying them to block TGF-β signaling have showed promise in reversing these effects 79.

Therapy evasion is also influenced by physical impediments. Collagen and hyaluronic acid make up the thick extracellular matrix (ECM) of GBM, which prevents CAR T-cell penetration 80. Furthermore, CAR T-cell distribution to the tumor location is restricted by the BBB. To get around these challenges, methods like directing CAR T-cells to release hyaluronidase or other ECM-degrading enzymes directly into the tumor or ventricular space have been used 81. The susceptibility of GBM cells to CAR T-cell-induced apoptosis is also decreased by inherent resistance mechanisms, such as the overexpression of anti-apoptotic proteins like Bcl-2 and the down regulation of death receptors like Fas. CAR T-cells can be modified to include pro-apoptotic signaling domains or mixed with small-molecule inhibitors that target anti-apoptotic pathways in order to address this 82. Furthermore, by changing CAR T-cell function and encouraging the release of substances like VEGF that block their action, tumor hypoxia in GBM worsens immune suppression. It has been suggested that one possible remedy is to use medications that modify hypoxia, such HIF inhibitors, or to engineer CAR T-cells to withstand hypoxia 83.

Innovative strategies are needed to overcome these evasion mechanisms, such as the creation of "armored" CAR T-cells that can withstand immunosuppressive signals, dual-targeting CAR T-cells that can address antigen heterogeneity, and combination therapies that use immune checkpoint inhibitors or chemoradiation 84. Additionally, CRISPR-Cas9 and other gene-editing technologies are being used to increase CAR T-cell resistance to the physical and immunological obstacles of GBM. These developments have the potential to increase CAR T-cell therapy's effectiveness in treating this difficult tumor type 85. Thus, there is an urgent need for innovative treatment approaches.

**Figure 6:**
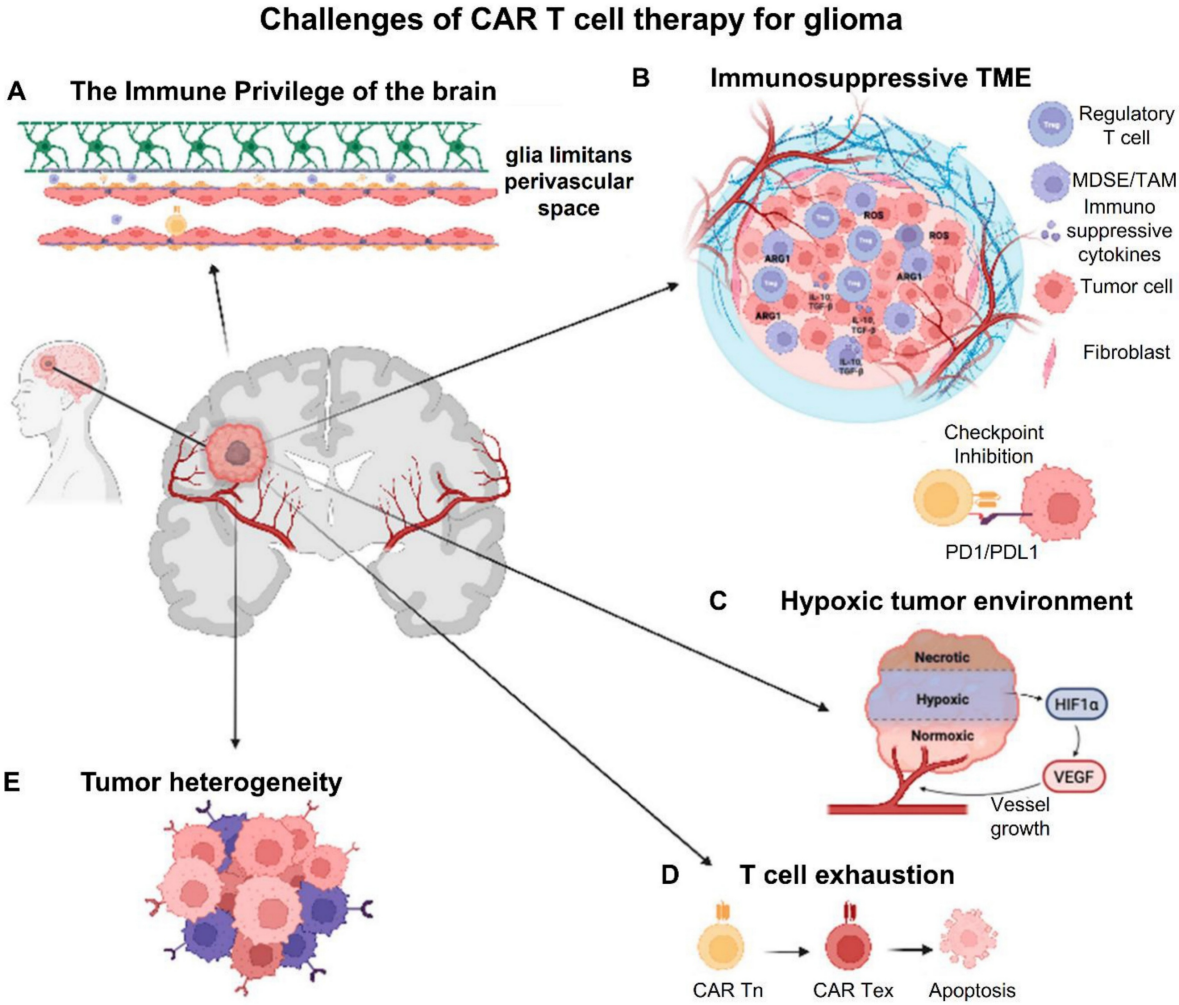
GMB CAR-T-cell therapy's difficulties. **(A)**. The brain's immune privilege creates biochemical and physical obstacles to CAR-T-cell homing. The blood-brain barrier is one of these barriers, and it is made up of pericytes buried in the capillary basement membrane, astrocyte end-feet that cover the capillary, and endothelial cells of the capillary wall. **(B)**. Pro-tumoral myeloid cells, immunosuppressive cytokines/chemokines, and checkpoint molecules are all components of the immunosuppressive TME, which works to prevent CAR-T-cell activation and effectiveness. **(C)**. In GBM, hypoxia produces an unfriendly environment that significantly restricts CAR-T cells' access to oxygen and nutrients. **(D)**. CAR-T-cell fatigue is caused by prolonged activation and antigen exposure. **(E)**. Target antigen selection and CAR construct design are significantly hampered by inter- and intra-tumoral heterogeneity. Reused under Creative Commons Attribution License (CC BY) 86.

In the study of Montoya *et al.* (2024), they outline current obstacles to the success of CAR-T-cell therapy for GBM, including the immune privilege of the CNS parenchyma, tumor heterogeneity, T-cell exhaustion, the cold (immunosuppressive) microenvironment, and local and systemic immunosuppression. In order to overcome the present obstacles in GBM CAR-T-cell therapy, they also discuss the advancements made in the creation of next-generation CAR-T cells and cutting-edge, unique techniques including low-intensity pulsed focused ultrasound **(Figure 6)** 86.

## CAR T Cells and Immune Exhaustion

Another major obstacle to the effectiveness of CAR T-cell treatment for GBM is immune fatigue. This condition happens when the suppressive TME, metabolic stress, and extended antigen exposure cause T-cells, including CAR T-cells, to become increasingly less effective 87, 88. Suboptimal treatment results from exhausted CAR T-cells' inability to multiply, release cytokines, and eliminate tumor cells. Enhancing the longevity and effectiveness of CAR T-cell treatment in GBM requires an understanding of and capacity to overcome immune fatigue 89. Chronic antigen stimulation is one of the main causes of CAR T-cell depletion in GBM. CAR T-cells are constantly activated in tumors like GBM, where antigens are always available. This results in the overexpression of inhibitory receptors such PD-1, TIM-3, LAG-3, and CTLA-4. These receptors contribute to functional fatigue by reducing T-cell activation 90, 91. For instance, research has demonstrated that CAR T-cells that target EGFRvIII in GBM gradually express more PD-1 and TIM-3, which is associated with decreased anticancer efficacy. Combining immune checkpoint inhibitors like anti-PD-1 or anti-CTLA-4 antibodies with CAR T-cell therapy has showed potential in addressing this. Preclinical studies showed that CAR T-cell activity and persistence are improved by inhibiting PD-1 signaling, which leads to improved tumor suppression 92, 93.

CAR T-cell depletion is also significantly aided by the immunosuppressive TME in GBM. High concentrations of immunosuppressive cytokines, such TGF-β and IL-10, which prevent T-cell activation and encourage fatigue, are a hallmark of GBM 94. To further reduce the immune response, TGF-β, for example, stimulates the development of regulatory T-cells (Tregs) and inhibits the generation of pro-inflammatory cytokines by CAR T-cells. To combat this, scientists have created armored CAR T-cells that are designed to withstand TGF-β's actions. Because these CAR T-cells carry a dominant-negative TGF-β receptor, they can continue to operate even when TGF-β levels are high. These armored CAR T-cells showed enhanced antitumor activity and persistence in the TME in preclinical studies 95, 96.

CAR T-cell fatigue is also influenced by metabolic stress in the TME. By consuming glucose and oxygen, GBM produces a hostile metabolic environment that deprives CAR T-cells of nutrients 97. GBM is characterized by hypoxia, which makes tiredness worse by causing hypoxia-inducible factors (HIFs) to be expressed, which hinders T-cell activity. One intriguing method to reduce fatigue is to engineer CAR T-cells to flourish in hypoxic environments. For instance, CAR T-cells that have been altered to produce proteins that stabilize HIF-1α have demonstrated enhanced functioning and survival in hypoxic conditions 98, 99. Cytokine support is another strategy to fight fatigue. CAR T-cells that are modified to release pro-inflammatory cytokines, such as IL-12 or IL-15, can attract and activate additional immune cells while improving their own survival and functionality. It has been demonstrated that IL-15, in particular, promotes memory T-cell production, which increases CAR T-cell persistence and decreases fatigue. CAR T-cells co-expressing IL-15 outperformed conventional CAR T-cells in tumor control and sustained their cytotoxic activity over time in a preclinical GBM model 100.

## Challenges and Improvements in CAR T Therapy for GBM

Because of its physical limitations, immunesuppressive microenvironment, and heterogeneity, GBM poses special difficulties for CAR T-cell treatment. Targeting many antigens to address the tumor's variety is one significant advance 101. GBM cells frequently defy treatment by downregulating or losing antigen expression, even if CAR T-cells that target single antigens like EGFRvIII initially show promise. Multi-specific CAR T-cells that target combinations like EGFRvIII, IL-13Rα2, and HER2 have been created to combat this, and preclinical studies have shown that they are more effective. Improving CAR T-cell infiltration is another crucial improvement 102. T-cell delivery and motility are restricted in GBM by the BBB and the thick extracellular matrix (ECM). While CAR T-cells have been engineered to release ECM-degrading enzymes like hyaluronidase, which has improved their capacity to infiltrate the tumor, direct intratumoral or intraventricular injection has demonstrated efficacy in avoiding the BBB 103.

**Figure 7:**
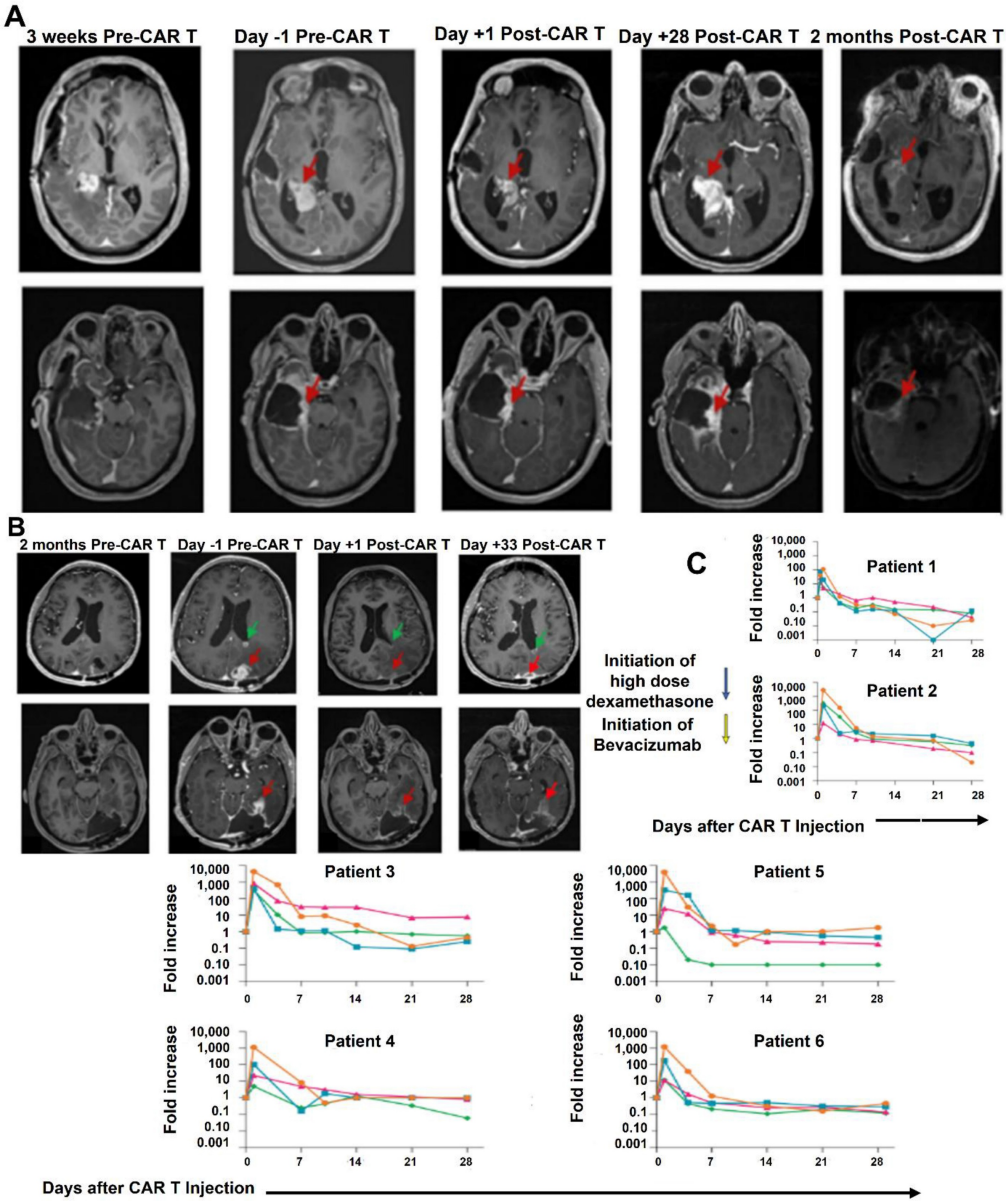
Multifocal rGBM regression upon intraventricular administration of CART-EGFR-IL13Rα2 cells (dose level 1). **(A)** Patient 2: A posterior mesial temporal lobe nodule (2.7 × 1.9 × 2.7 cm) grew significantly over three weeks. Post-CAR T therapy, nodule size and enhancement initially decreased but later expanded by day +28. Without further treatment, subsequent scans revealed regression of the nodule and stabilization of resection cavity enhancement.** (B)** Patient 1: Axial gadolinium-enhanced T1-weighted images revealed a solidly enhancing left parieto-occipital nodule (red arrow) and a necrotic nodule near the left lateral ventricle ependyma (green arrow) that developed over two months. Post-CAR T cell infusion, the prominent lesion receded within 24 hours (red arrow). By day +33, improvement continued with central necrosis at the lesion site, though spatial enlargement of the left occipital resection cavity (red arrow) was noted and surgically removed. Treatment-related alterations were seen in 90% of tissue samples. **(C)** Cytokine data (IFNγ, IL-2, TNFα, IL-6) showed peaks within 1-4 days post-CAR T infusion, reverting to baseline by day 14. High-dose dexamethasone was initiated 10-24 hours post-injection Adapted with permission from reference 104 Copyrights 2024, SPRINGER NATURE.

Bagley *et al*. (2024) stated in his study that recurrent rGBM relics a major unmet medical necessity, with an average general survival of less than 1 year. Here, they describe the first six rGBM patients treated with intra-thecal delivery of bivalent CAR T cells that target IL13Rα2 and the EGFR in a phase 1 study. Safety and figuring out the maximum tolerable dosage were the study's main goals. The incidence of manufacturing defects and objective radiographic response (ORR) based on modified Response Assessment in Neuro-Oncology criteria are secondary objectives that were reported in this interim analysis. At the time of therapy, all six patients had multifocal, progressing illness. The administration of CART-EGFR-IL13Rα2 cells was linked to early-onset neurotoxicity in both dose levels 1 (1 × 107 cells; n = 3) and 2 (2.5 × 107 cells; n = 3). This was most consistent with immune effector cell-associated neurotoxicity syndrome (ICANS), which was treated with high-dose dexamethasone and anakinra (anti-IL1R). A dose-limiting toxicity (grade 3 anorexia, tiredness, and widespread muscular weakness) occurred in one patient at dosage level 2. All six patients showed reductions in tumor size and enhancement at early magnetic resonance imaging timepoints, but none of them satisfied ORR criteria. All six patients had significant CAR T cell abundance and cytokine production in the cerebrospinal fluid, according to exploratory endpoint assessments. When combined, these first-in-human findings show that CART-EGFR-IL13Rα2 cells are initially safe and bioactive in rGBM **(Figure 7)** 104.

**Figure 8:**
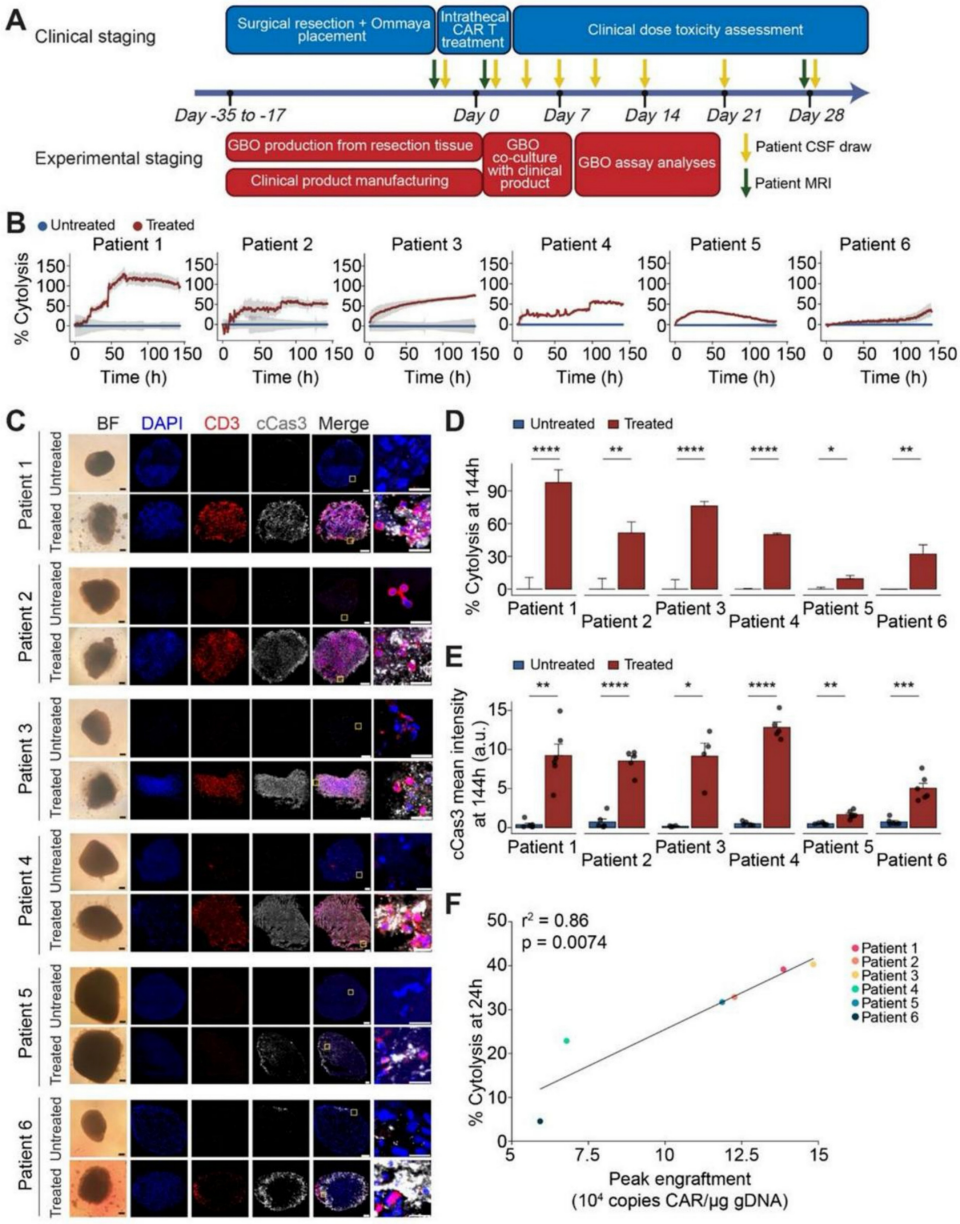
Significant tumor cell cytolysis was seen upon co-culturing GBM organoids with patient-matched CAR-T cells, and this was connected with clinical CAR-T cell engraftment. **(A)** Scheduling for correlative GBO tests conducted concurrently with patient care in clinical trial NCT05168423. **(B)** Using the Axion Maestro Cellular Impedance platform, a time-course examination of GBO cytolysis with co-cultivation of autologous CAR-T cells over 144 hours demonstrates that patient CAR-T cells (Treated) had a lower impedance of GBOs than untreated GBOs (Control). **(C)** Sample bright field (BF) and immunofluorescence pictures for DAPI, CD3, and cleaved caspase 3 (cCas3) in treated and untreated GBOs show that the treated GBOs underwent cCas3+ tumor cell death in addition to CD3+ T cell infiltration. **(D)**. identical dataset as that in (B). The values show the mean ± S.E.M **(E)** quantification of cCas3 immunostaining in autologous CAR-T cell treated and untreated GBOs confirmed that the results seen in (B) and (D) were caused by apoptotic cell death. **(F)** A correlation between the peak CAR-T cell engraftment levels in the respective patients and the average cytolysis values in the treated GBOs at 24 hours. Adapted with permission from reference 105 Copyrights 2024, ELSEVIER.

According to a study by Logun *et al*. (2024) patient-derived tumor organoids have been used for preclinical research and disease modeling, but they are seldom used in real time to help understand patient treatment responses in clinical settings. In a first-in-human, phase 1 study utilizing dual-targeting CAR T cells (EGFR-IL13Rα2 CAR-T cells) in patients with recurrent GBM, they showed early effectiveness signs. They examined six sets of GBM organoids (GBOs) produced from patients who received the identical autologous CAR-T cell products as those who were treated in phase 1 research. They discovered that the degree of CAR-T cell engraftment identified in patients' cerebrospinal fluid (CSF) was connected with the decrease of target antigen and cytolysis of tumor cells in GBOs that resulted from CAR-T cell therapy. Moreover, GBOs' cytokine release patterns over time mirrored those of patient CSF samples **(Figure 8)**. The study's conclusions point to a novel trial design and GBOs as a useful tool for evaluating CAR-T cell bioactivity in real time and gaining knowledge about the effectiveness of immunotherapy 105.

Improved resilience is demonstrated by armored CAR T-cells, which are designed to either release pro-inflammatory cytokines like IL-12 or to withstand TGF-β stimulation. By avoiding fatigue, CAR T-cell therapy's effectiveness has been further increased when combined with immune checkpoint inhibitors such anti-PD-1 or anti-CTLA-4 antibodies.

Furthermore, treating tumor hypoxia, a characteristic of GBM, has the potential to enhance results. In addition to inhibiting CAR T-cell persistence, hypoxia also triggers the production of VEGF and other molecules that support tumor life. These effects can be lessened by modifying CAR T-cells to operate in hypoxic environments or by pairing them with medications that inhibit hypoxia-inducible factors (HIFs) 106. Enhancing CAR T-cell durability and persistence is also necessary for long-lasting therapeutic benefits. In preclinical settings, CAR T-cells have demonstrated improved survival and prolonged activity through the incorporation of cytokine-secreting characteristics like IL-15 or co-stimulatory domains like 4-1BB or OX40. Another intriguing option is combination therapy; for example, CAR T-cell therapy combined with chemotherapy or radiation has been shown to have synergistic benefits. For instance, radiation improves immune cell infiltration and antigen presentation, both of which increase the effectiveness of CAR T-cells 107. Lastly, to increase specificity and lessen off-target effects, sophisticated CAR designs are being investigated, such as logic-gated or dual-antigen CAR T-cells. By better differentiating tumor cells from healthy tissue, these designs help CAR T-cells reduce collateral harm 108, 109. These novel approaches have the potential to overcome the drawbacks of CAR T-cell treatment for GBM, notwithstanding the difficulties. CAR T-cell therapy has the potential to revolutionize the treatment of this debilitating illness by targeting tumor heterogeneity, boosting persistence within the immunosuppressive environment, and improving infiltration 110.

Liang *et al*. (2023) has said in his study that innovative CAR-T cell designs and regional delivery. To prevent tumor antigen heterogeneity, for example, the structure of multi-antigen-targeted CAR-T cells can enhance CAR-T accumulation in tumor TME and eradicate many tumor cells. Additionally, several generations of advancements in the structure and production of CAR-T cells have increased effectiveness and persistence when combined with an immune modifier and one or more stimulating domains. However, the clinical survival advantage of single CAR-T cell treatment is minimal. Combination treatments have added to the therapeutic paradigm in comparison to solo CAR-T cell therapy. Based on several outstanding studies, combinatorial treatment approaches enhance the effectiveness of CAR-T cells by controlling the tumor microenvironment, enhancing the CAR structure, directing the CAR-T cells to the tumor cells, and reversing the tumor-immune escape mechanisms. They also offer a promising approach to GBM.

**Figure 9:**
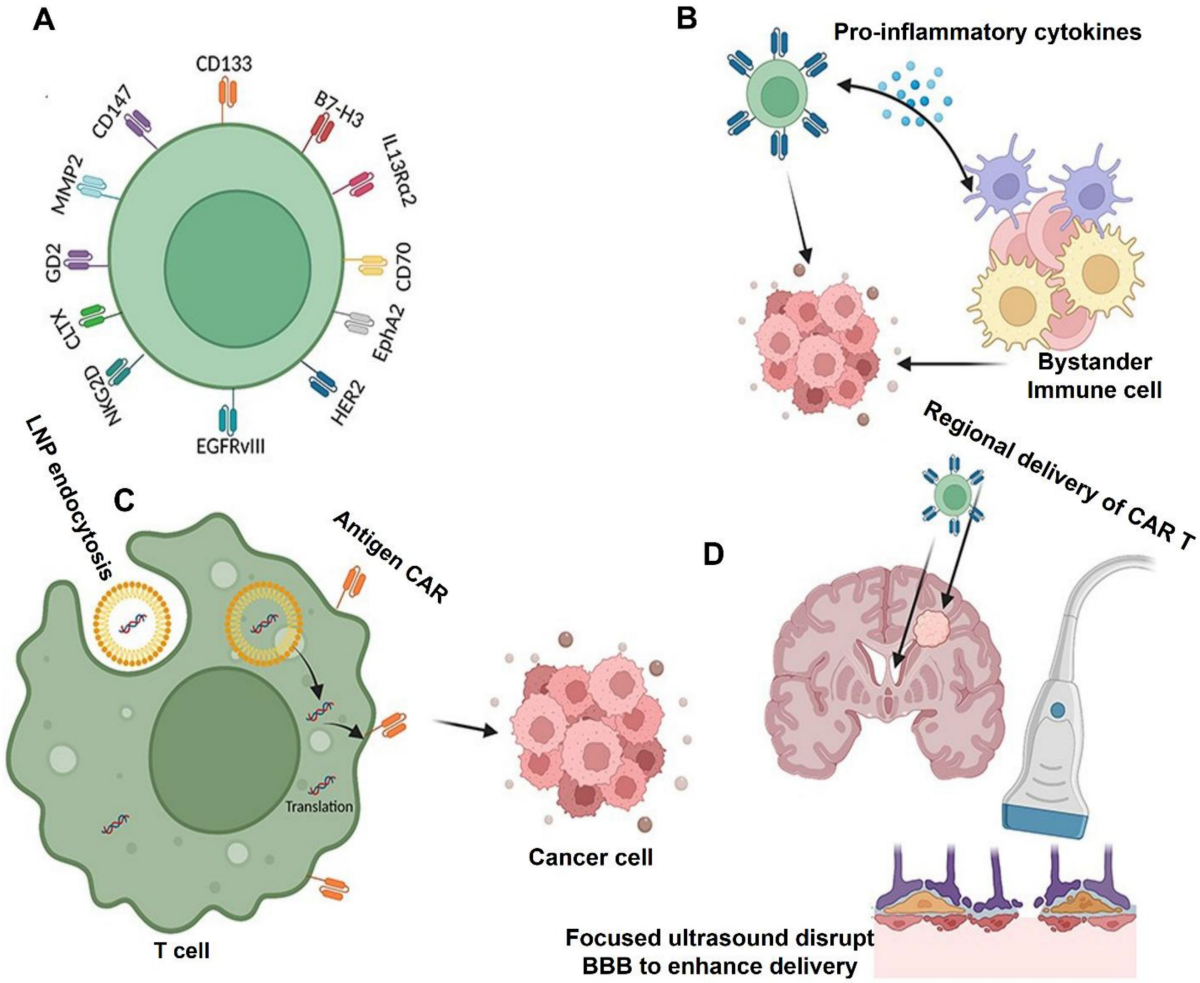
Techniques to improve GBM CAR-T cell function. **(A)** Using new target antigens to design CAR-T cells for GBM; **(B)** Using bystander immune cells to boost antitumor activity; **(C)** Multifunctional mRNA-based CAR-T cells; **(D)** Using focused ultrasound and regional CAR-T cell delivery may affect immune cell activation and CAR-T delivery. Reused under Creative Commons Attribution License (CC BY) 111.

Additionally, promising outcomes have been shown in preclinical and clinical trial samples when effective medicines are combined with CAR-T cells. This has sparked interest in investigating the anticancer activity of combination therapy. Although GBM therapy still has several limitations, the most common target antigens for GBM CAR-T cell treatments include EGFRvIII, HER2, and IL-13 Rα2. Advanced CAR-T cells have recently been created with new targets such CD133, B7-H3, CLTX, and NKG2DLs. Additionally, investigator-engineered T cells overcome the clinical issue of antigen-negative tumor escape following adoptive cell therapy by secreting dendritic cell (DC) growth factor Fms-like tyrosine kinase 3 ligands (Flt3L) to stimulate the endogenous DCs and overcome tumor antigen heterogeneity **(Figure 9)** 111.

According to Li *et al*. (2025), CAR T cell treatment is a very successful immunotherapy for hematological cancers, but it is still difficult to use against the majority of solid tumors. Here, a new synergistic combination treatment against solid tumors was presented, combining CAR-T cell therapy with drug-free triboelectric immunotherapy. By combining the effects of electrostatic breakdown with triboelectrification, a triboelectric nanogenerator (TENG) was created that can produce pulsed direct current. To power the triboelectric device, the TENG can produce up to 30 pulse direct-current peaks in a single slide, with a peak current output of around 35 μA. Pulsed direct-current stimulation caused tumor cells to die immunogenically (with a 35.9% survival rate), which aided in the development of dendritic cells, sped up the presentation of antigen to CAR-T cells, and improved the systemic adaptive immune response. Moreover, triboelectric immunotherapy reconfigured the tumor immunosuppressive microenvironment, decreased regulatory T cell differentiation, and boosted M1-like macrophage polarization, all of which improved the ability of CAR-T cells to eliminate about 60% of the solid tumor mass of NALM6. Notably, the combination therapy did not raise the burden of double-medication on patients, which is noteworthy given that triboelectric immunotherapy is a safe and effective drug-free anticancer method 112.

### Targeting Alternative Antigens and Multi-Targeting Strategies

A key tactic to increase the effectiveness of CAR T-cell treatment in GBM, a very diverse and adaptable tumor, is to target different antigens. Conventional methods frequently concentrate on well-known antigens unique to GBM, including EGFRvIII. Antigen escape is the term for the process by which many GBM cells avoid treatment by downregulating or ceasing to express their major antigens 113. In order to overcome this restriction, scientists are now concentrating on finding and destroying a wider range of tumor antigens and investigating antigen combinations to improve treatment results 114.

**Figure 10:**
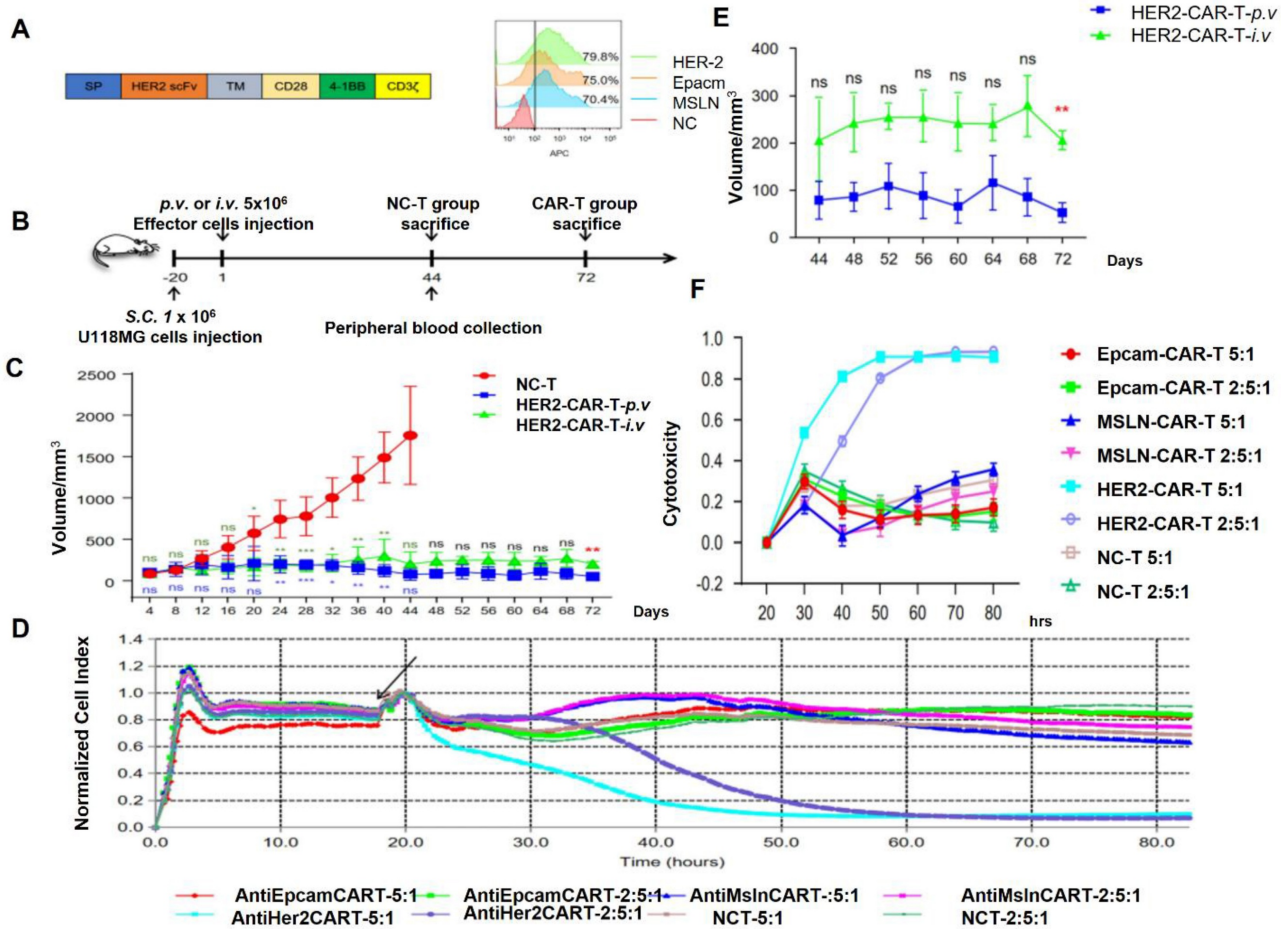
** (A)** HER2 CAR-T transgenic schematic design. Flow cytometry was used to evaluate the expression of HER2, EpCAM, and MSLN. APC streptavidin (1:1000) was added after CAR-T cells had been treated with biotinylated anti-HER2, anti-MSLN, and anti-EpCAM. **(B)** Outline of the experiment. Three sets of NCG mice were created, each consisting of six mice. Mice received a subcutaneous injection of 1 × 106 U118MG cells. Mice carrying xenografts were given 5 × 106 NC-T and CAR-T cells once the tumors were well-established on day 20. **(C)** Every four days, the size of the tumor was measured. The NC-T against HER2-CAR-T-p.v. and NC-T versus HER2-CAR-T-i.v. analyses were shown in blue and dark green fonts, respectively. **(D)** The RTCA test was used to assess the cytotoxicity of CAR-T cells against solid tumor cell lines. Analysis was done on the cytotoxicity of HER2-, EpCAM-, and MSLN-CAR-T and NCT cells against U118MG cells for 80 hours at an E: T of 5:1 and 2.5:1. **(E)** The effector cell addition time is indicated by the black arrow. **(F)** E:T ratios and death rates on U118MG cells under various effector cell conditions are compared. Three separate experiments are represented by the data, which are displayed as the mean ± SD of triplicates. Reused under Creative Commons Attribution License (CC BY) 124.

IL-13Rα2 is a potential alternative antigen that is seldom present in normal tissues but is overexpressed in many GBM cells. Significant anticancer effectiveness has been demonstrated by CAR T-cells that target IL-13Rα2 in preclinical models and early clinical studies. A patient with recurrent GBM experienced partial regression after receiving intratumor delivery of IL-13Rα2-specific CAR T-cells 115. Notwithstanding these early achievements, not all GBM cells express IL-13Rα2, underscoring the necessity of multi-targeting strategies. HER2 is another alternative antigen that is expressed in subsets of GBM but is best recognized for its involvement in breast cancer **Figure 10**. Preclinical models have demonstrated the effectiveness of HER2-targeted CAR T-cells, and early-phase clinical studies have yielded positive outcomes. However, to reduce off-tumor damage, careful planning is required due to HER2 expression in normal tissues 116.

B7-H3, an immune checkpoint protein present on tumor cells and vasculature, and GD2, a ganglioside expressed in GBM and other malignancies, are further potential antigens. In preclinical models, GD2-targeted CAR T-cells have demonstrated potential, especially when combined with immune checkpoint inhibitors 117. Similarly, because B7-H3 is selectively expressed in tumor tissues, CAR T-cells that target it have shown strong anticancer effectiveness with a lower chance of off-target consequences. These antigens are part of an expanding target repertoire that can be used to enhance treatment 118. To improve specificity and lessen antigen escape, researchers are also investigating logic-gated CAR designs and multi-antigen CAR T-cells 119.

Despite these developments, concerns like antigen heterogeneity and the possibility of target antigens being expressed similarly in healthy tissues still exist 120, 121. In order to get around these problems, scientists are merging CAR T-cell therapy with antigen screening tools like proteomics and single-cell sequencing to find new, tumor-restricted antigens 122. These methods enable the identification of targets particular to each patient and the creation of customized CAR T-cell treatments based on the distinct antigenic profile of every tumor 123.

### Targeting Multiple Antigens

Because of its considerable intratumoral heterogeneity, GBM is notoriously difficult to treat with single-target CAR T-cell treatments 125. Targeting a single antigen might result in antigen escape, a phenomenon in which tumor cells down regulate or stop expressing the targeted antigen. Tumor cells in GBM frequently display a variety of antigen expression patterns. Targeting several antigens at once has become a viable method to address this issue, increasing the effectiveness of CAR T-cell therapy and lowering the risk of treatment failure 126.

The use of CAR T-cells to target EGFRvIII, a mutation-specific antigen present in GBM, is a well-established illustration of the necessity of multi-antigen targeting 127. While first research showed some effectiveness, limited responses resulted from the heterogeneity of EGFRvIII expression in malignancies, as many tumor cells were completely devoid of this antigen 128, 129. Dual-target CAR T-cells, which can identify EGFRvIII and another GBM-associated antigen like IL-13Rα2 or HER2, have being investigated by researchers as a potential solution to this problem. Due to their ability to identify and eradicate a wider variety of tumor cells, these dual-targeted CAR T-cells have demonstrated improved anticancer effectiveness in preclinical models 130. Using "OR-gated" CAR T-cell designs, which enable a single CAR T-cell to autonomously identify several antigens, is an additional strategy 131. For instance, tumor cells expressing either antigen can be bound and killed separately by CAR T-cells that are designed to target both IL-13Rα2 and B7-H3, an immunological checkpoint protein produced by GBM cells. This approach lessens the possibility that the tumor population will be dominated by antigen-negative escape variants. Similarly, by addressing the heterogeneity of antigen expression inside GBM, CAR T-cells targeting combinations such as GD2, HER2, and EGFRvIII have shown enhanced effectiveness in experimental models 132.

Researchers are looking on "AND-gated" CAR T-cell designs, which need the simultaneous presence of two antigens for activation, in addition to OR-gated CAR T-cells. By lowering the possibility of off-target effects in healthy tissues that only express one of the antigens, this dual recognition strategy improves specificity. For example, a CAR T-cell that is AND-gated may be designed to activate only when it comes into contact with EGFRvIII and IL-13Rα2 on the same tumor cell. These innovations raise the accuracy of tumor targeting while simultaneously enhancing safety 133. The use of CAR T-cells targeting a combination of HER2 and CD133, the latter of which is a marker linked to glioma stem cells (GSCs), has also been studied in preclinical and early clinical trials. GSCs are a particularly hostile subset of GBM cells that are linked to treatment resistance and tumor recurrence. By focusing on both markers, these CAR T-cells may target differentiated tumor cells and stem-like cells at the same time, decreasing the likelihood that the tumor will grow again. For instance, compared to single-antigen CAR T-cells, dual-targeted CAR T-cells that recognize both EGFRvIII and IL-13Rα2 have demonstrated improved antitumor effectiveness because they can eradicate a variety of tumor cell types. Furthermore, logic-gated CAR T-cells have strong tumor-killing potential while lowering the possibility of harming healthy cells since they need two different antigens to activate. For example, in order to activate cytotoxic activities and ensure precise targeting "AND-gated" CAR T-cell design may need to recognize IL-13Rα2 and HER2 simultaneously 134.

Researchers are using cutting-edge technologies like tandem CARs and bifunctional CARs to improve multi-antigen targeting even more. Tandem CARs are designed to identify several antigens at once by combining two different antigen-binding domains into a single construct 135. Bifunctional CARs, on the other hand, are made to increase the immune response against cancers that express a variety of antigens by producing cytokines or other immune-activating chemicals in response to antigen recognition. A bifunctional CAR that targets both EGFRvIII and IL-13Rα2 may, for instance, also release IL-12 to attract and activate more immune cells in the tumor microenvironment 136. There are still difficulties in spite of these developments. As the number of targets rises, so does the difficulty of developing and producing multi-antigen CAR T-cells. To prevent unintentional toxicity, it is crucial to balance the specificity and affinity of each antigen-binding domain 137. Furthermore, determining the best antigen combinations necessitates a thorough comprehension of the molecular landscape of GBM, which might differ greatly from patient to patient. Proteomics and single-cell RNA sequencing are two methods being used to identify antigen expression patterns and guide the creation of customized CAR T-cell treatments for specific malignancies 138-140.

**Figure 11:**
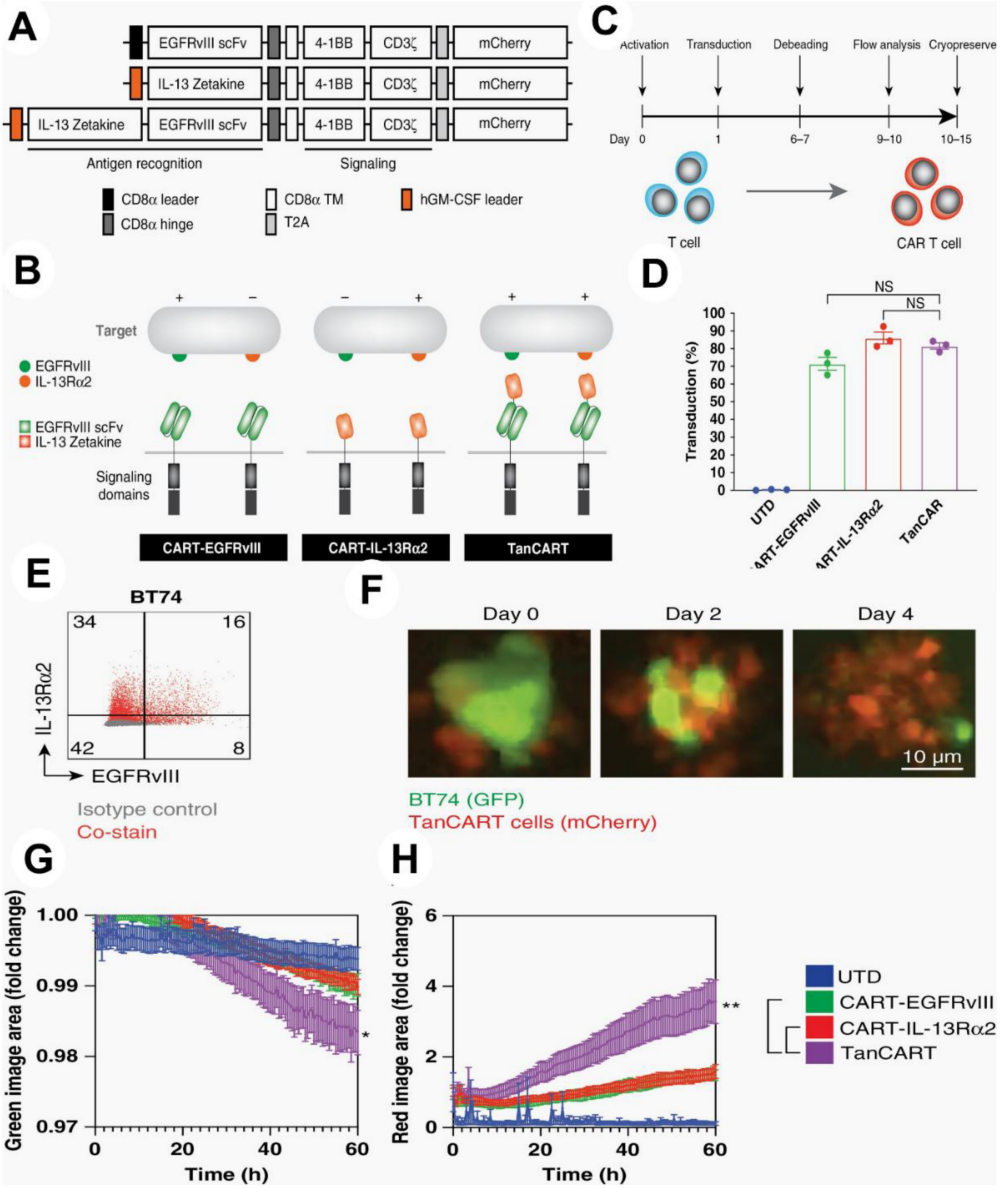
**(1)** TanCART cell design and production. **(A)** EGFRvIII and IL-13Rˑ2 were the targets of single- and dual-specific CARs. **(B)** Second-generation constructions for TanCART, CART-EGFRvIII, and CART-IL-13Rˑ2 are shown. **(C)** Diagram showing the schedule for CAR T cell manufacturing. **(D)** Mean CAR transduction effectiveness in three healthy donors' primary human T cells. The data is displayed as mean ± SD. **(E)** TanCART cells grow in longitudinal experiments *in vitro* and are effective against heterogeneous GBM PDX. EGFRvIII and IL-13Rα2 baseline surface expression levels in the GBM PDX, BT74. **(F)** Typical pictures of TanCART cells co-cultured with BT74 neuropsheres during a cytotoxicity test with a 1:1 effector-to-target ratio. **(G)** Quantification of entire green image area for live-cell study of BT74 neurosphere lysis by UTD, CART-EGFRvIII, CART-IL-13Rα2, and TanCART cells. **(H)** Total red image area as a measure of effector cell proliferation. The data are displayed as mean ± SEM and were measured in triplicate. At least two distinct normal donors participated in the experiments again, and the outcomes were comparable. Reused under Creative Commons Attribution License (CC BY) 141.

To prove the effectiveness of this strategy against a variety of tumor types, they used a number of orthotopic preclinical models, including patient-derived xenografts, in addition to conventional immunological tests. *In vitro*, tandem CAR T cells showed increased cytotoxicity against patient-derived brain tumor cultures as well as other diverse GBM populations (P <.05). In orthotopic murine models of heterogeneous GBM, including patient-derived xenografts, dual antigen engagement via the tandem construct was required to elicit long-term, full, and persistent responses, in contrast to CAR T cells that target single antigens (P <.05). They show that TanCART works well against a variety of brain tumor types. The development of multi-specific CAR T cells for the treatment of GBM and other malignancies is further supported by other studies **(Figure 11)** 141.

### Optimizing CAR T-cell design

CAR T-cell depletion is a major barrier to GBM treatment, however creative solutions are being created to overcome this issue. Researchers want to improve the longevity and effectiveness of CAR T-cells by optimizing CAR T-cell design. By adding more activation signals, co-stimulatory domains like 4-1BB (CD137) or OX40 (CD134) to CAR constructions improve T-cell survival and lessen fatigue. In preclinical GBM models, CAR T-cells including 4-1BB domains have shown better antitumor activity and durability than those containing CD28 domains alone. To avoid persistent overstimulation and lessen fatigue, dual-switch CAR T-cells which alternate between activation and rest phases are also being created 142. There are still difficulties in spite of these developments. Designing successful treatments requires keeping an eye on fatigue indicators and comprehending the dynamic interplay between CAR T-cells and the TME 143. Proteomics and single-cell RNA sequencing are examples of emerging technologies that shed light on the molecular mechanisms behind tiredness and allow for the creation of more focused therapies 144.

**Figure 12:**
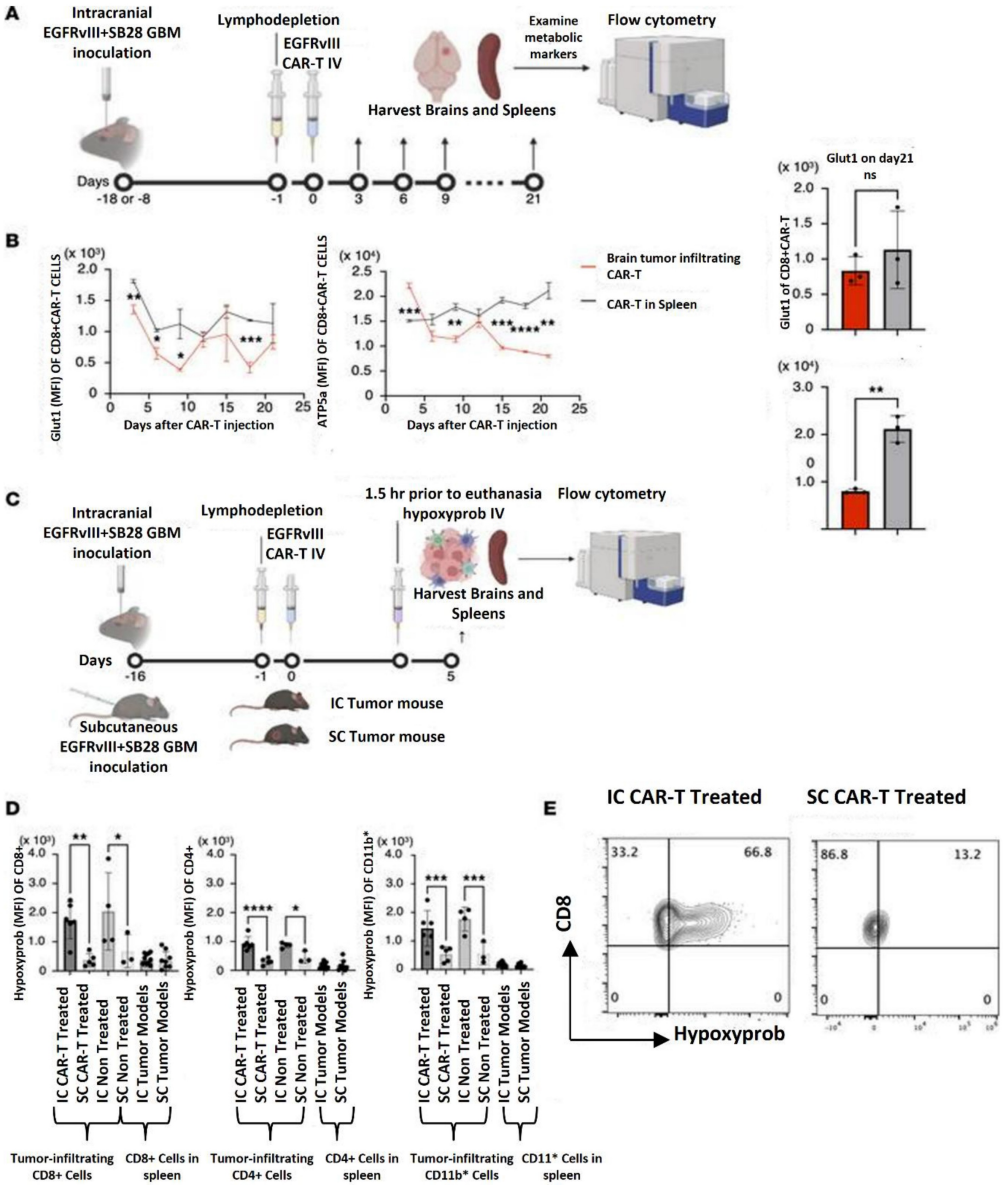
In the hypoxic GMB microenvironment, decreased OXPHOS activity is linked to CAR-T cell exhaustion. **(A)** The experimental setup for assessing CAR-T cells that infiltrate GMBs. IV stands for intravenous. **(B)** Glioma-infiltrating CD8^+^ CAR-T cells' longitudinal alterations in Glut1 and ATP5a, indicators of the glycolytic pathway and OXPHOS, respectively (left panels). Mean fluorescence intensity (MFI) of CD8^+^ CAR-T cells isolated from the tumor (red) and spleen (gray) on day 21 shows the expression of Glut1 (top right) and ATP5a (bottom right). **(C)** The setup for *in vivo* analysis of hypoxic conditions. The hypoxyprobe is taken up by CD8^+^ (left), CD4^+^ (middle), or CD11b^+^ (right) leukocytes that infiltrate the spleens or intracranial tumor model (IC) or subcutaneous tumor model (SC) of mice with GMBs. The data is displayed as mean ± SD. *P < 0.05; **P < 0.01; ***P < 0.001, ****P < 0.0001 by 1-way ANOVA followed by Tukey's multiple comparison test **(D)** or by unpaired, 2-tailed t test (B). **(E)** Representative histograms (IC or SC tumors in CAR-treated animals) demonstrating that CD8^+^ BILs, but not CD8^+^ CAR-T cells isolated from SC tumors, stain positively with hypoxyprobe. Reused under Creative Commons Attribution License (CC-BY) 145.

Hatae *et al*. (2024) used the experimental system of anti-EGFRvIII CAR-T cells obtained from EGFRvIII CAR-T-transgenic mice that they previously created to examine the metabolic state inside the tumor microenvironment. They can conduct extended tests with great scientific rigor thanks to this approach, which makes it possible to create a huge number of CAR-T cells with uniform quality. After intravenous (i.v.) infusion into syngeneic C57BL/6J mice with intracerebral SB28 tumors expressing human epidermal growth factor variant III (SB28 hEGFRvIII), they assessed the metabolic state of CAR-T cells. One intravenous infusion of 3 × 106 anti-EGFRvIII CAR-T cells was given to the animals. Up to 21 days following injection, a cohort of three mice was slaughtered every three days. The GMB tissues were then removed, and flow cytometry was used to assess the metabolic state of the CAR-T cells that had infiltrated the brain tumor. The CD8^+^ CAR-T cells recovered from the GMB tissues and those produced from the spleen did not vary substantially in their expression levels of the glycolytic marker glucose transporter 1 (Glut1). Conversely, the GMB -infiltrating CD8^+^ CAR-T cells' expression levels of ATP synthase (ATP5a), a hallmark of OXPHOS, steadily declined with time, but the spleen-derived CD8^+^ CAR-T cells' expression levels did not exhibit similar declining patterns. Additionally, CD4^+^ CAR-T cells showed comparable results. They postulated that the hypoxic GMB microenvironment may be the cause of the reduced OXPHOS activity in CAR-T cells since OXPHOS is a biological mechanism that uses oxygen in the internal mitochondria to make a significant quantity of ATP. they i.v. delivered anti-EGFRvIII CAR-T cells to mice with day 16 SB28 mEGFRvIII tumors in the brain or subcutaneous area in the right flank of syngeneic C57BL/6J mice in order to test this idea. Because luciferase expression in the mEGFRvIII version is more consistent than in SB28 hEGFRvIII, they employed SB28 mEGFRvIII in *in vivo* investigations (data not shown). Mice were given hypoxyprobe, an agent that is preferentially absorbed by hypoxic cells, five days later. The GMB tissues and spleens were harvested 1.5 hours following the hypoxyprobe infusion. Regardless of CAR-T cell treatment, CD8^+^, CD4^+^, and CD11b^+^ populations in the intracerebral GMB tissue were substantially more hypoxic than those in subcutaneous tumors, according to flow cytometric analyses of CD8^+^, CD4^+^, and CD11b^+^ cells extracted from these organs **(Figure 12)** 145.

**Figure 13:**
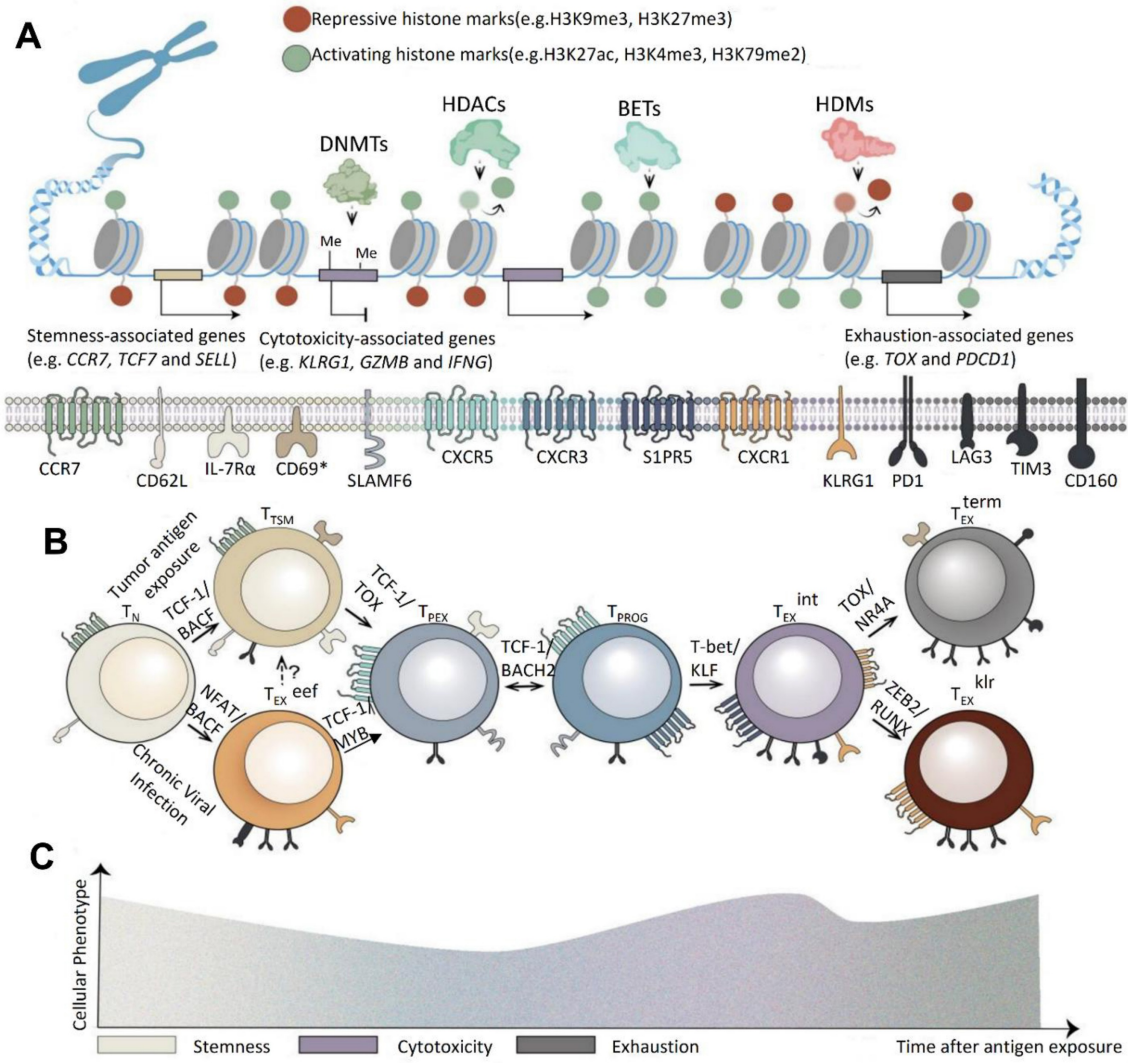
Genetic and Phenotypic Diversity in T Cell Exhaustion Progression. **(A)** Exhausted T cell subsets exhibit distinct epigenetic profiles. Naïve T cells (TN_NN​) are characterized by closed chromatin configurations and repressive DNA methylation patterns maintained by DNMT3a. Chronic exposure to tumor antigens increases chromatin accessibility near stemness-associated genes in TN_NN​ cells, enabling the expression of transcription factors such as TCF-1. Additionally, epigenetic modifiers, including writers, erasers, and readers, regulate activation-related chromatin regions (ACRs) linked to cytotoxicity in S1PR5 intermediate exhausted T cells (T_EX_int ​) and T_EX_KLR effector exhausted T cells (T_EX_ klr​), as well as exhaustion-related ACRs in TOX T_EX_ cells. **(B)** The progression of T cell exhaustion is orchestrated by a sequential hierarchy of transcription factors. Lineage-defining transcription factors influence chromatin accessibility and activate networks of lineage-specific genes, guiding the differentiation of TN_NN​cells into tumor-specific memory T cells (T_TSM_) within tumor-draining lymph nodes (TdLNs). In chronic viral infections, a distinct subset of early effector T cells (T_EX_eff​) emerges under NFAT regulation, displaying high levels of exhaustion-associated surface molecules. These cells subsequently differentiate into IL-7R/SLAMF precursor exhausted T cells (T_PEX_​), a process controlled by MYB. T_PEX_ cells further evolve into progenitor exhausted T cells (T_PROG_), marked by CXCR3 upregulation for migration into the tumor microenvironment (TME). Transcription factors such as KLF, TOX, and ZEB2 respectively drive the differentiation of T_EX_int ​, T_EX_, and T_EX_klr subsets. **(C)** Prolonged antigen stimulation alters the dominant phenotypes of T cells. Notably, CD69 expression is observed in tissue-resident T_TSM_cells in TdLNs and in liver-derived T_EX_​cells, highlighting their tissue-specific residency. Reused under Creative Commons Attribution License (CC-BY) 146.

According to Xiong *et al*. (2024), CAR-T cell therapy, a type of adoptive cell therapy (ACT), has emerged as a promising approach in cancer treatment. FDA-approved CAR-T treatments that target CD19 and B cell maturation antigen (BCMA) for hematological malignancies serve as proof of this. Even with these improvements, solid tumor results are still not ideal. T cell fatigue, which lowers the durability and functioning of CAR-T cells and causes recurrence rates of up to 75% in patients undergoing CD19 or CD22 CAR-T therapies for hematological malignancies, poses a serious threat to the efficacy of CAR-T therapy. Utilizing state-of-the-art single-cell sequencing and genomic engineering technologies is necessary to address this problem. Notably, early-stage T cell subsets exhibit little depletion because they are less affected by the tumor immune microenvironment (TIME). Because these memory-like T cells can differentiate into immune checkpoint blockade (ICB)-induced late-stage tumor-infiltrating lymphocytes (TILs) instead of functioning as completely cytolytic cells, they are essential for maintaining lasting therapeutic responses **(Figure 13)**. By finding therapeutic targets for modifying T cell behavior and offering biomarkers to improve patient selection, a better understanding of the cellular and molecular mechanisms causing T cell depletion may improve CAR-T treatment 146.

## Image-guided CAR-T therapy of GBM

In order to improve the accuracy and effectiveness of CAR T cell therapies, image-guided CAR T therapy incorporates real-time imaging modalities. This allows for the monitoring of T cell trafficking, tumor targeting, and therapeutic responses *in vivo* 147, 148. Making sure CAR T cells are efficiently trafficked to tumor locations is one of the main hurdles in treating GBM, especially in light of the BBB and the disease's spatially diffuse character. One potential approach is reporter gene imaging. For instance, reporter genes like sodium iodide symporter (NIS), enhanced green fluorescent protein (eGFP), or herpes simplex virus type 1 thymidine kinase (HSV-tk) can be inserted into CAR T cells. By interacting with certain imaging probes, including radiotracers or fluorescent dyes, these reporter genes enable the detection of CAR T cells through the use of imaging modalities such as near-infrared fluorescence (NIRF), single-photon emission computed tomography (SPECT), and positron emission tomography (PET) 149.

High sensitivity and the capacity to measure the distribution and persistence of CAR T cells throughout time are provided by PET imaging in particular. For example, preclinical research involved engineering CAR T cells to produce HSV-tk in order to target IL13Rα2, an antigen that is frequently overexpressed in GBM 150. Researchers were able to track the migration of CAR T cells into intracranial tumor locations and evaluate the effectiveness of treatment by delivering radiolabeled substrates that attach specifically to cells that express HSV-tk. This method helps improve dosing regimens based on real-time data and offers insightful information about the kinetics of CAR T cell delivery 151. Imaging methods are essential for assessing tumor responses and identifying lingering illness in addition to monitoring CAR T cells. Following CAR T cell treatment, photoacoustic and NIRF imaging have demonstrated promise in detecting any leftover tumor cells. Researchers can see how CAR T cells interact with tumor tissue and identify regions where tumor eradication is still partial, for instance, by labeling CAR T cells with a fluorophore 152.

The combination of MRI and CAR T cells modified with super paramagnetic iron oxide nanoparticles (SPIONs) is one innovative technique. By improving MRI contrast, these nanoparticles make it possible to monitor CAR T cells in great detail as they cross the BBB and enter the GBM microenvironment. The increase of SPION-labeled CAR T cells in tumor areas was precisely shown in a recent study that targeted EGFRvIII, a mutation common in GBM. Additionally, this strategy identified areas where CAR T cells were unable to enter, offering crucial information for enhancing delivery techniques. By ensuring that CAR T cells reach their targeted targets while reducing toxicity and off-target effects in non-tumor areas, it improves therapeutic accuracy 153. Furthermore, depending on observed responses, imaging-guided approaches enable real-time treatment protocol adjustments, such as changing cell dosages or combining treatments. Additionally, by linking CAR T cell location and persistence to patient prognosis, this method can aid in treatment outcome prediction 154.

Image-guided CAR T treatment is gradually approaching clinical use, although being mostly in the experimental and preclinical phases. This strategy might be further improved by combining cutting-edge machine learning algorithms with imaging modalities including PET, MRI, and fluorescence imaging 155. For example, artificial intelligence (AI) can detect biomarkers linked to the success or failure of therapy and forecast the behavior of CAR T cells by analyzing complicated imaging information. A thorough grasp of CAR T cell dynamics and GBM responses may also be possible with the integration of multiplexed imaging, which blends many imaging modalities on a single platform. Many of the obstacles presently restricting the effectiveness of CAR T cells in GBM should be removed with the inclusion of image-guided CAR T treatment as researchers continue to innovate. By increasing accuracy, lowering toxicity, and facilitating a customized approach to immunotherapy, this tactic has the potential to revolutionize the treatment of GBM 156.

MRI tracking plays a crucial role in image-guided CAR-T therapy for GBM by enabling real-time, non-invasive monitoring of CAR-T cell localization, tumor response, and potential adverse effects. MRI offers high-resolution imaging to evaluate therapy effectiveness and direct therapeutic modifications due to the difficulties with CAR-T cell trafficking and persistence inside the brain. Researchers can monitor CAR-T cell movement, infiltration, and distribution within the tumor microenvironment using sophisticated MRI methods such contrast-enhanced imaging and SPIO nanoparticle tagging. Clinicians can improve patient outcomes, optimize dosage regimens, and increase T-cell delivery accuracy while reducing off-target effects by combining MRI tracking with image-guided CAR-T treatment. This collaboration between immunotherapy and imaging has the potential to advance CAR-T therapy as a practical therapeutic option and break down treatment hurdles for GBM.

### MRI tracing

Magnetic resonance imaging (MRI) tracing has emerged as a critical technique in enhancing the accuracy and efficacy of CAR T cell treatment for GBM 157. Effective monitoring of CAR T cells within the brain is essential to comprehending their biodistribution, activity, and therapeutic effects since GBM is a very invasive and heterogeneous malignancy 158. With its excellent spatial resolution and non-invasive imaging capabilities, MRI tracing offers a useful platform for real-time CAR T cell monitoring, allowing physicians and researchers to maximize the therapeutic potential and delivery of these cells 159. SPIONs, which produce observable signal alterations in MRI scans, are used to mark CAR T cells in order to facilitate MRI-based monitoring. Because SPIONs are biocompatible and have a high T2-weighted contrast, they may be used to monitor cells inside the intricate structure of the brain. Following their infusion into patients, CAR T cells that have been loaded with SPIONs may be monitored as they move through the circulation, penetrate the BBB, and enter tumor locations 160.

Understanding the activity of CAR T cells in the highly immunosuppressive GBM microenvironment requires constant monitoring of their distribution and persistence, which is made possible by MRI tracing 161. In one research, SPION-labeled CAR T cells that were injected intracerebrally to get over the BBB were tracked using MRI. A handful of CAR T cells spread out into neighboring brain areas, but the bulk of them confined to the tumor location, according to the imaging. These findings demonstrate how useful MRI tracing is for detecting off-target effects and enhancing the accuracy of treatment plans. Moreover, to assess alterations in the tumor microenvironment, MRI tracing can be coupled with cutting-edge imaging methods like functional MRI (fMRI) 162. For example, after receiving CAR T cell therapy, researchers have evaluated changes in tumor perfusion and vascularity using fMRI and correlated these changes with the effectiveness of the treatment. This multimodal method helps guide changes in treatment regimens by offering a thorough understanding of how CAR T cells interact with GBM tissue.

In GBM mice models, this work labeled CD70 CAR T cells and monitored their *in vivo* migration and therapeutic activity using novel MegaPro nanoparticles (MegaPro-NPs); to keep an eye on the identified CAR T cells, researchers used magnetic particle imaging and repetitive MRI images 163. According to the study, the nanoparticles enhanced the contrast of the MRI signal, allowing for accurate tracking of the biodistribution of T cells. The outcomes showed that the CAR T cells effectively homed to the tumor sites of GBM and remained there throughout time. Crucially, the imaging revealed areas of the tumor that were not well penetrated, indicating that multimodal imaging may help direct the development of CAR T cell delivery strategies in clinical situations 164. Tumor oxygenation during CAR T-cell treatment using fluorine-19 (19F) MRI in conjunction with perfluorocarbon (PFC) probe biosensors. Following the infusion of CAR T cells, this method allowed for the non-invasive, real-time monitoring of the oxygen status within GBM tumors. According to the imaging, the tumor microenvironment's oxygenation levels significantly increased as a result of CAR T cells targeting GBM, indicating that the hypoxic conditions necessary for tumor development had been disturbed. By following metabolic and environmental changes in tumors, the results demonstrated the usefulness of 19F MRI as a technique for both tracking the biodistribution of CAR T cells and evaluating their therapeutic efficacy 165.

In a recent study SPIONs were used to mark immune cells, including CAR T cells, in GBM models in order to provide high-resolution imaging of their movement and activation. The study explained how MRI can give researchers non-invasive, long-term views into how immune cells behave in the brain, allowing them to evaluate the effectiveness of their homing and potential therapeutic benefits. It also covered issues with signal specificity, labeling efficiency, and applying these techniques to human research 166. The study assessed the immune response to CAR T-cell treatment in GBM models using a combination of contrast-enhanced MRI and PET imaging. While PET offered quantitative information on metabolic activity, MRI was used to assess structural alterations in malignancies. The study showed that after CAR T cell treatment, MRI could identify minute changes in tumor vascularization and edema. PET results that showed an increase in immune cell activity within treated tumors were connected with these data. This dual-modality method guided improvements in dosage and administration techniques and offered a thorough evaluation of treatment effectiveness 167.

**Figure 14:**
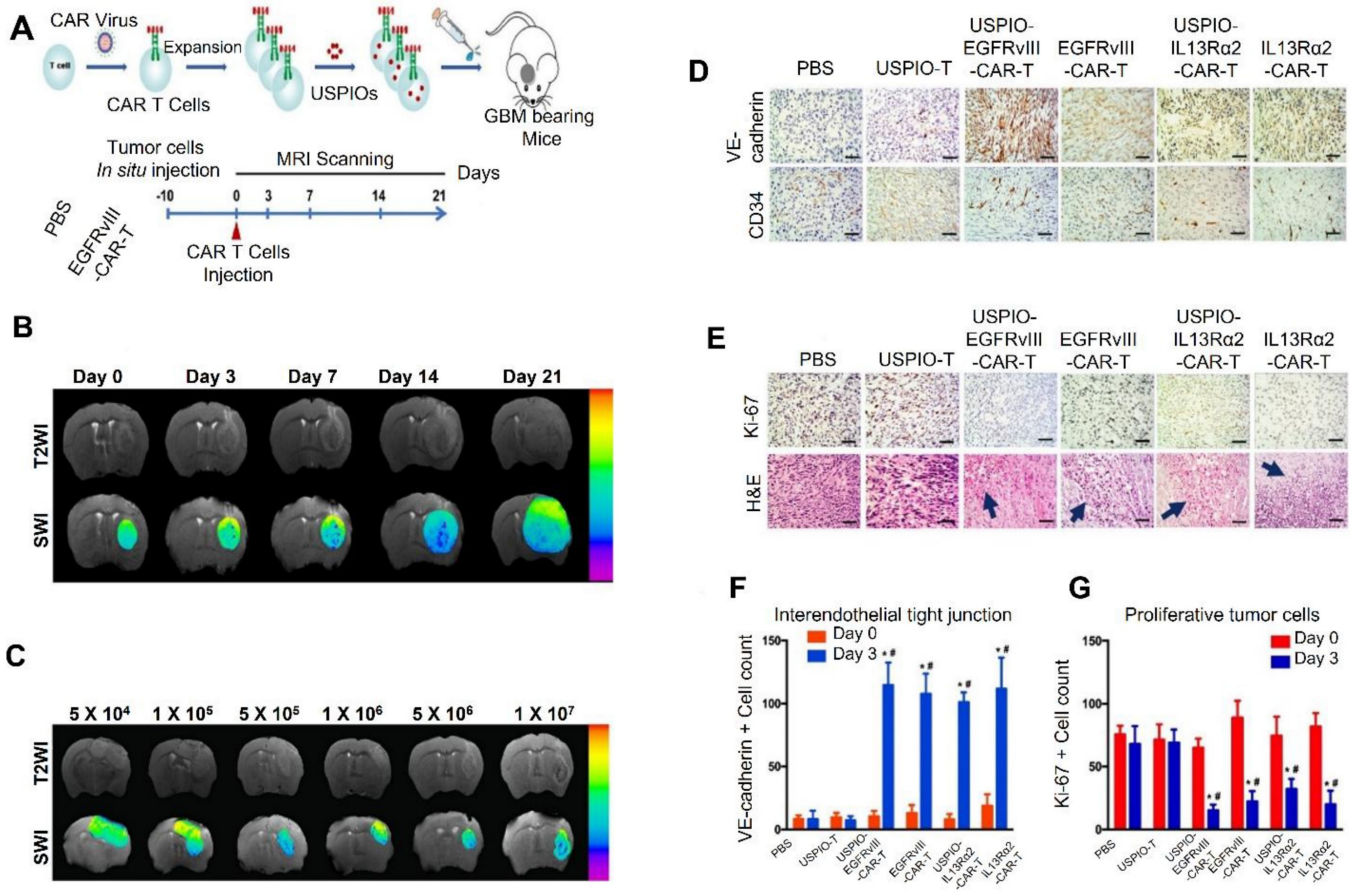
**(A)** Sequential MRI images of GBM xenografts and CAR T-cell treatment were part of the experimental design. **(B)** GBM-bearing mice were given USPIO-labeled EGFRvIII CAR T cells at baseline and on days 3, 7, 14, and 21 following the injection of the T2WI and SWI MRI scans. These areas showed up on SWI MRI imaging as deep blue and purple patches. **(C)** Using different dosages of USPIO-labeled EGFRvIII CAR T cells, T2WI and SWI MRI images were also obtained seven days after therapy. **(D)** On day three following the injection of USPIO-labeled CAR T cells, immunostaining showed an increase in intercellular tight junctions inside the tumor microenvironment, as evidenced by VE-cadherin-positive cells. **(E)** On day three after therapy, H&E staining revealed regions of regional **(F)** VE-cadherin-positive cells in tumors were quantitatively analyzed, and the results, which were displayed as mean ± SD **(G)** The mean ± SD of five separate studies was used to quantify Ki-67-positive cells, which showed considerably lower proliferative activity in tumor. Adapted with permission from reference 168. Copyrights 2021, ELSEVIER.

Xie *et al*. in 2021 reported in his study that one potential method for treating solid tumors is CAR T-cell therapy. Understanding the invasion, durability, and therapeutic benefits of CAR T cells requires the use of *in vivo* cell monitoring tools. In this situation, magnetic resonance imaging (MRI) becomes an important technique that offers high-resolution cellular imaging using specific probes and offers a multitude of biological information on solid tumors. Ultra-small superparamagnetic iron oxide particles (USPIOs), which are glucose-coated amino alcohol derivatives of nanoparticles, they used in his work to mark CAR T cells for non-invasive monitoring of their infiltration and persistence in GBM. The CAR constructions specifically targeted IL-13 receptor subunit alpha 2 (IL13Rα2) and human epidermal growth factor receptor variant III (EGFRvIII). When used at the right quantities, USPIO labeling did not affect the cytotoxicity or biological characteristics of CAR T cells. From day 3 to day 14 following the injection of USPIO-labeled CAR T cells, MRI utilizing susceptibility-weighted imaging showed steadily rising hypointensity signals in GBM models. The presence of CAR T cells and nanoparticles in matched tissue slices was verified by histopathological examination. Additionally, perfusion and diffusion at day three after therapy, MRI showed increased water diffusion and decreased vascular permeability, which were consistent with immunostaining tests' findings of decreased tumor cell growth and increased intercellular connections. This study demonstrates the early therapeutic benefit of CAR T cells by highlighting an efficient imaging method for monitoring them in GBM models. The assessment and improvement of CAR T-cell treatments for solid tumors can be influenced by these findings **Figure 14** 168.

**Figure 15:**
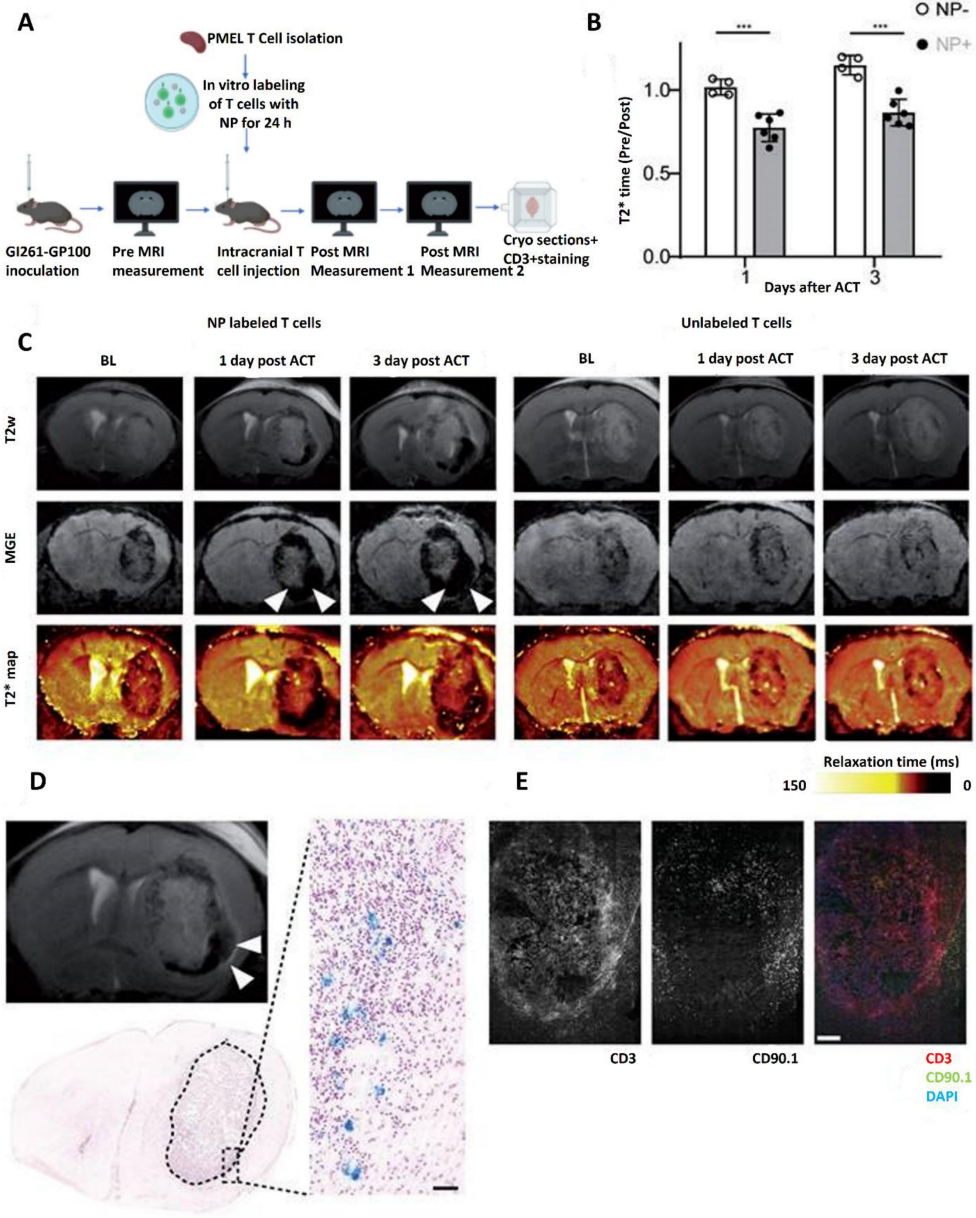
**(A)** Experimental protocol for intratumoral T cell injections and MRI data is shown schematically. **(B)** T2* maps are used to compare the tumor relaxation times of iron oxide NP-labeled T cells versus unlabeled T cells at 1 and 3 days after intratumoral ACT. In comparison to baseline (pre-ACT) results, relaxation times were adjusted. **(C)** MRI data showing the mouse brain following intratumoral ACT with iron oxide NP-labeled and unlabeled T cells, including T2-weighted images, multi-gradient echo (MGE) sequences, and T2* maps. **(D)** Prussian blue staining utilizing iron oxide NP-labeled T cells to compare with MRI results following ACT. **(E)** Confocal microscopy imaging of sections co-stained with DAPI, CD3+, and CD90.1+ for immunohistochemical examination. Reused under Creative Commons Attribution License (CC BY) 169.

According to Hunger *et al.* (2023) one kind of intrinsic brain tumor that is particularly resistant to immunotherapies, such as immune checkpoint inhibitors, is GMBs. A potential development in GMB immunotherapy is adoptive cell treatments (ACT), such as T cell receptor (TCR)-transgenic therapies and CAR T cell therapies that target glioma-associated antigens. Non-invasive imaging methods for tracking these adoptively transplanted T cells' homing to the GMB microenvironment are still severely lacking, though. This was addressed by employing ultrasmall iron oxide nanoparticles (NPs), which may be identified by magnetic resonance imaging (MRI) utilizing certain sequences such as T2* mapping. To effectively mark human and murine TCR-transgenic and CAR T cells with iron oxide nanoparticles *ex vivo*, a methodology was created. Using transmission electron microscopy (TEM) and flow cytometry, labeling effectiveness and T cell function were evaluated. High-field MRI at 9.4 Tesla was used to observe tagged T cells *in vivo* after adoptive transfer, and the imaging results were compared to three-dimensional models of cleared brains produced by light sheet microscopy (LSM). The results showed that NPs are efficiently absorbed into T cell subcellular cytoplasmic vesicles, attaining high labeling efficiency without sacrificing antigen-specific cytotoxicity, cell survival, proliferation, or cytokine production. Longitudinal MRI monitoring showed very sensitive intratumoral T cell infiltration in a mouse GBM model. Furthermore, T2* imaging revealed that the spatial distribution of T cells in the tumor microenvironment (TME) was predictive of the effectiveness of treatment. Incomplete coverage was linked to treatment resistance, while robust T cell infiltration and even distribution were associated with tumor response. The possibility of employing iron oxide nanoparticles to non-invasively track adoptive T cell treatments for GBMs is demonstrated by this work. These imaging techniques may make it easier to monitor intratumoral T cell dynamics and function as a predictor of treatment effectiveness **Figure 15** 169.

### PET imaging for tracing CAR T

Real-time, non-invasive tracking of biological processes within the body is made possible by the advanced molecular imaging method known as Positron Emission Tomography (PET) imaging. PET imaging is essential for tracking the location, persistence, and activity of CAR T cells during GBM CAR T-cell immunotherapy 170. This capacity is essential for determining the effectiveness of treatments, streamlining treatment plans, and reducing side effects. PET imaging is a vital tool for assessing the activity of these modified immune cells, and CAR T-cell therapy has demonstrated promise in treating GBM, a famously aggressive and treatment-resistant brain tumor. Radiolabeling CAR T cells with isotopes like fluorine-18 (¹⁸F), copper-64 (⁶⁴Cu), or zirconium-89 (⁸⁹Zr) is a popular PET imaging technique. The identification of CAR T cells is made possible by the conjugation of these isotopes to molecules that attach to them 171.

In a study with anti-EGFR CAR T cells labeled with zirconium-89 in GBM mice models, the cells' effective localization to tumor sites and persistence over a two-week period were demonstrated by PET imaging, which also linked their presence to better treatment results. These findings highlight PET imaging's capacity to forecast CAR T-cell therapy effectiveness and improve its use 172. Reporter gene imaging, in which CAR T cells are modified to express certain genes, including sodium iodide symporter (NIS) or herpes simplex virus thymidine kinase (HSV-tk), is another intriguing technique. These genes enable CAR T cells to preferentially accumulate PET-compatible substrates, such as radiolabeled fluorothymidine. HSV-tk-expressing CAR T cells were used for ¹⁸F-FEAU PET imaging in one noteworthy study, which offered real-time insights into their growth and tumor invasion. This method is especially well-suited for clinical applications as it provides the benefit of long-term monitoring without necessitating the direct tagging of cells 173.

CAR T cell tracking has also been investigated using immuno-PET, a method that combines PET imaging and the specificity of monoclonal antibodies. CD19-targeted PET probe to assess the location of CAR T cells in GBM models. This technique made it possible to precisely identify the location and infiltration of CAR T-cells into tumor locations, which provided important information for evaluating the effectiveness of treatment. The precision of CAR T-cell tracking *in vivo* is improved by immuno-PET's capacity to take advantage of antigen-specific interactions. When employing CAR T cells to treat GBM, PET imaging has a number of significant uses. It makes it possible for researchers to verify that CAR T cells target tumors, especially when it comes to getting beyond the BBB 174. Through the use of PET imaging GD2-targeted CAR T cells could localize to GBM tumors, confirming their efficacy in overcoming the brain's defenses. As demonstrated by a clinical trial (NCT04099797) that employed zirconium-89-labeled CAR T cells to predict treatment responses in patients with recurrent GBM, PET imaging may also be utilized as a predictive tool by linking the presence of CAR T cells in the tumor with clinical outcomes.

Furthermore, after delivery, CAR T-cell survival and persistence may be tracked over time using PET imaging. This is essential for comprehending how long the immune response lasts and figuring out what affects treatment results. By seeing CAR T-cell increase in non-target tissues, it can also aid in the detection of off-target toxicity, reducing the possibility of immune-related side effects 175.

Although its encouraging potential, there are still issues with PET imaging in CAR T-cell therapy for GBM, including the need to improve resolution to differentiate CAR T cells from surrounding tissues, optimize radiotracers for specificity and durability, and integrate PET imaging with other modalities like MRI to obtain complementary insights. However, CAR T immunotherapy for GBM has advanced thanks in large part to PET imaging. It makes it possible to precisely track CAR T cells, assess the effectiveness of treatment, and spot side effects, all of which give vital information for improving patient outcomes and therapeutic strategy 176.

**Figure 16:**
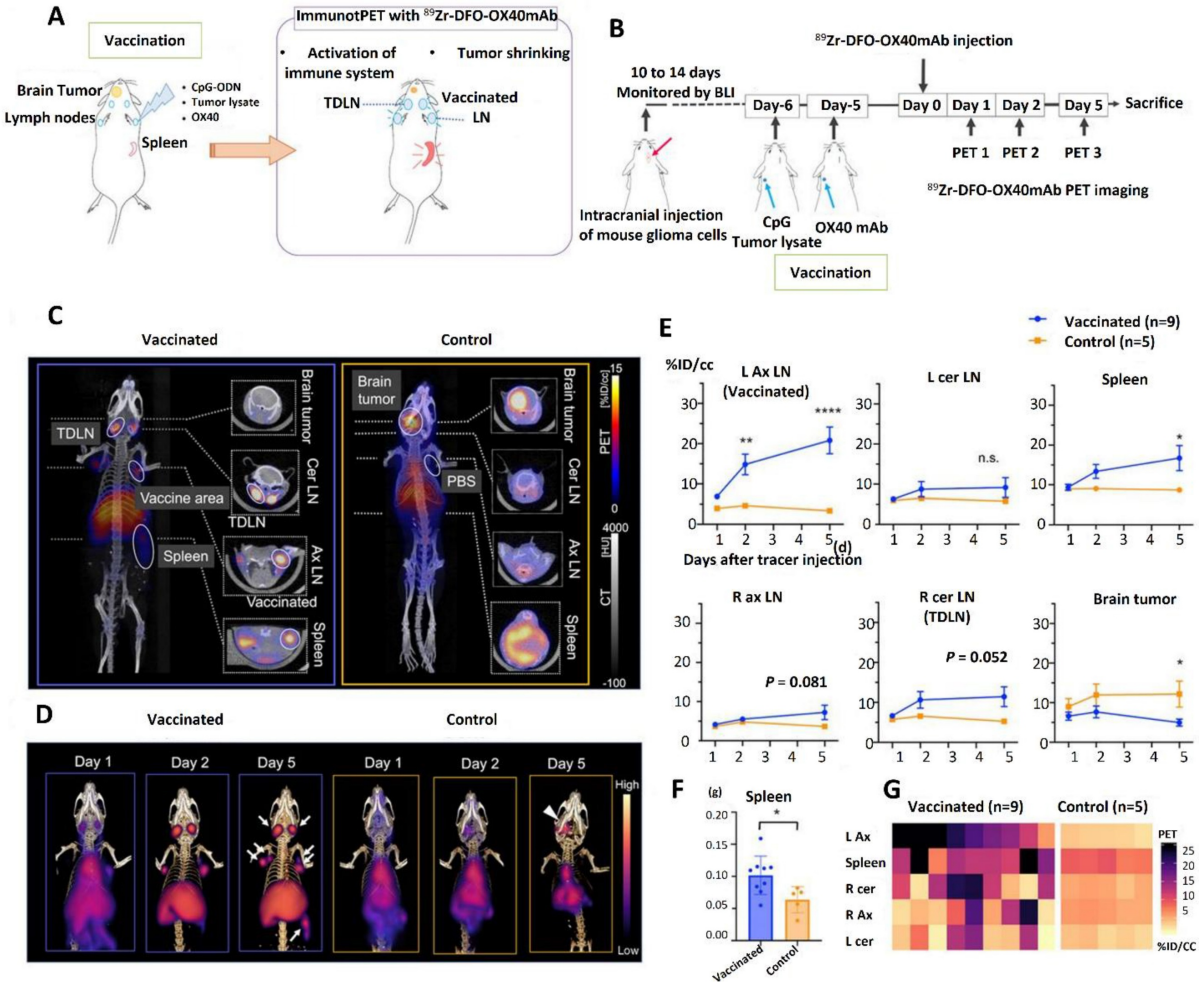
**(A)** According to the study's basic framework, mice having GBMs in the right hemisphere were given immunization treatment to the left shoulder. ImmunoPET imaging using ⁸⁹Zr-DFO-OX40 mAb was utilized to evaluate therapy effects and observe immune system activity, namely in the lymph nodes close to the immunization site and tumor-draining lymph nodes (TDLNs). **(B)** GBM cells were injected intracranially into the right hemisphere as part of the experimental protocol. Bioluminescence imaging (BLI) was then used to monitor the tumor over a period of 10-14 days. Five and six days before the injection of ⁸⁹Zr-DFO-OX40 mAb, respectively, the left shoulder received subcutaneous injections of CpG in combination with tumor lysate and OX40 mAb. On days 1, 2, and 5 following the administration of the radiotracer, sequential PET scans were performed. **(C)** Day 5 representative PET/CT pictures showed the differences between the control and vaccinated animals. Tracer uptake was observed in many lymphatic organs, including the spleen, the right cervical lymph node (TDLN), and the left axillary lymph node (vaccinated site), according to maximum intensity projection (MIP) pictures of vaccinated mice. Control mice, on the other hand, showed clear tumor signals in the right hemisphere but no activated lymph nodes. The exact sites of tracer accumulation in the designated areas were validated by axial fusion pictures. **(D)** As early as day 2, three-dimensional PET/CT scans of the vaccinated mice showed obviously active spleens and lymph nodes, but by day 5, control animals had huge tumors in their right brain. **(E)** PET signal time-course analysis showed that vaccinated mice had much higher radiotracer uptake than controls, especially in the spleen (P = 0.020) and left axillary lymph node (P < 0.0001) on day 5. There were almost significant variations in the amount of tracer accumulation between the right cervical lymph node (TDLN) and the right axillary lymph node. Additionally, on day 5, vaccinated mice showed much less PET signals in their brain tumors (P = 0.042). **(F)** When compared to controls, the vaccinated mice's spleen volume was much larger, suggesting splenomegaly (P = 0.019). **(G)** The left axillary lymph node (vaccinated location) had the greatest mean PET signal in vaccinated mice, followed by the spleen, right cervical lymph node (TDLN), and right axillary lymph node, according to a correlogram of ⁸⁹Zr-DFO-OX40 mAb signals on day 5. The observed differences in PET signal strength between vaccinated and control groups were highly significant, with P-values ranging from <0.05 to <0.0001. Adapted with permission from reference 177. Copyrights 2021, American Association for Cancer Research.

According to Nobashi *et al.* in a 2021 study one intriguing treatment option for GBM and other oncological cancers is immunotherapy. But as of this now, there are no reliable instruments or biomarkers to assess systemic immune responses in GBM patients receiving immunotherapy in a thorough manner. The potential of OX40, a costimulatory molecule mostly expressed on activated effector T cells involved in the elimination of cancer cells, as a PET imaging biomarker to measure and track immunotherapy responses was investigated in this work. A subcutaneous vaccination strategy utilizing CpG oligodeoxynucleotide, OX40 monoclonal antibody (mAb), and tumor lysate at a distant location was developed using a mouse orthotopic GBM model. In order to evaluate the location of T cells in activated lymphoid organs in connection to treatment results, this approach was created to activate T cells distally. Using *in vivo* PET/CT imaging in conjunction with the in-house-developed radiotracer ⁸⁹Zr-DFO-OX40 mAb, OX40-positive T cells were identified. In addition to the lymph nodes close to the vaccination site (mean uptake of 20.8% ID/cc), immunoPET imaging with ⁸⁹Zr-DFO-OX40 mAb showed a significant and specific OX40-positive signal in the spleen (16.7% ID/cc) and tumor-draining lymph nodes (11.4% ID/cc). The immunization approach resulted in a large number of responsive T cells and a considerable reduction in tumor signal after only one cycle of therapy when the tumors were small (less than 10⁶ photons/sec/cm²/sr as determined by bioluminescence imaging). These results demonstrate how cancer vaccination may be used as a treatment for GBM and how useful immunoPET imaging is for tracking T-cell activation and treatment effectiveness **Figure 16** 177.

A developing and potent technique for monitoring and assessing the dynamics of CAR T-cell treatments, especially in the treatment of GBM, is radionuclide-based molecular imaging 178. This imaging method provides real-time information on the location, distribution, and persistence of CAR T-cells within tumor locations by using radiolabeled molecules to observe and measure biological events *in vivo*. Optimizing CAR T-cell therapy in clinical settings requires non-invasive, high-sensitivity imaging, which is made possible by the integration of radionuclides into CAR T cells or targeting molecules. Radionuclide-based molecular imaging can be employed in CAR T immunotherapy to track the activity of CAR T cells in the brain, an organ that poses many difficulties because of the BBB 179.

**Figure 17:**
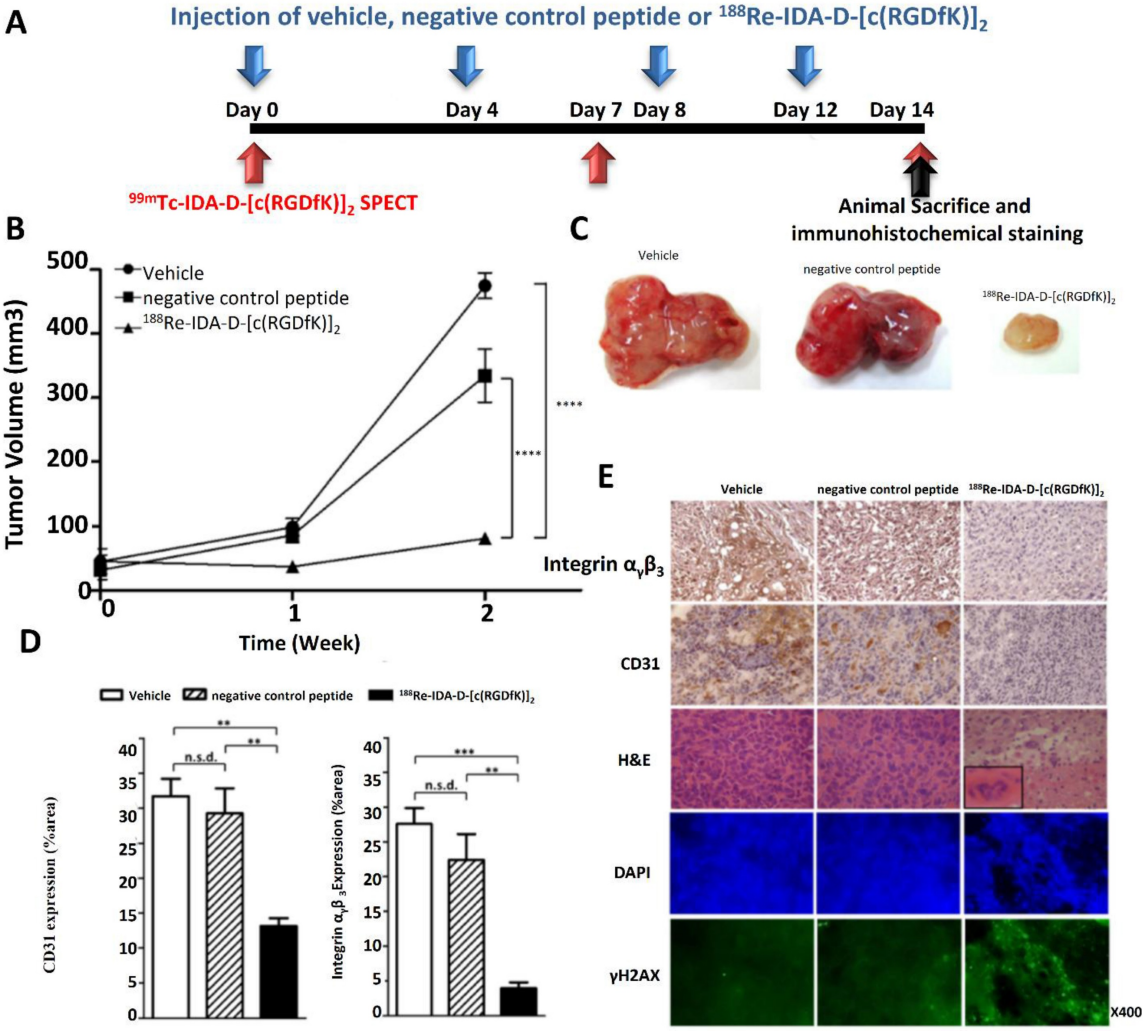
Using tumor-bearing mice, the anti-angiogenic properties of 188Re-IDA-D-[c(RGDfK)]2 (11.1 MBq) were assessed in U87-MG xenografts. After being split up into three groups, these mice were given 188Re-IDA-D-[c(RADfK)]2, a negative control peptide (188Re-IDA-D-[c(RGDfK)]2), or the vehicle for two weeks (n = 4 per group). **(A)** A schematic illustration of the treatment strategy was provided. **(B)** At different intervals, the tumor volume was tracked. **(C)** The macroscopic appearance of the dissected tumors in each of the three groups was shown in representative photographs. **(D)** Following the two-week therapy, the percentage area of microvessels and integrin αvβ3 positivity in tumors were evaluated for each experimental group. **(E)** To assess DNA damage, tumor slices were subjected to immunofluorescence imaging using DAPI and γH2AX, as well as immunohistochemistry analysis utilizing anti-αvβ3, anti-CD31, and H&E staining. ANOVA and Bonferroni post hoc tests (**p < 0.01, ***p < 0.001, ****p < 0.0001) were used to statistically evaluate the data; NSD denoted no significant difference. Reused under Creative Commons Attribution License (CC-BY) 180.

Lee *et al*. (2021) stated in his study that tumor angiogenesis-related receptors, such integrin αvβ3, are useful indicators for cancer detection and treatment because GBM, a very aggressive brain tumor, is linked to strong angiogenesis. This study used 188Re-IDA-D-[c(RGDfK)]2 at a dosage of 11.1 MBq to examine the therapeutic potential of peptide receptor radionuclide treatment (PRRT). The results showed that, in comparison to vehicle-treated controls, PRRT considerably decreased tumor volume in U87-MG xenografts, with an 81% reduction. Using 99mTc-IDA-D-[c(RGDfK)]2 single-photon emission computed tomography (SPECT) in conjunction with tumor volume measurements, a quantitative *in vivo* investigation of anti-angiogenic effects was accomplished. Furthermore, the combination of PRRT and temozolomide (TMZ) improved therapeutic efficacy, leading to a 93% decrease in tumor volume, which was significantly higher than the outcome of either treatment alone. Interestingly, histological research revealed that this combo therapy produced better results even when TMZ was given at half the usual dosage. Given that integrin αvβ3 is a crucial target for tumor angiogenesis-directed theranostics, the findings imply that integrin-targeted PRRT in conjunction with TMZ may be a viable approach for the successful treatment of GBM **Figure 17** 180.

### Multimodal *In Vivo* Tracking

A key strategy for expanding the use of CAR T cells in the treatment of GBM is multimodal *in vivo* tracking. The BBB, tumor heterogeneity, and an immunosuppressive milieu are some of the particular difficulties that GBM poses as an aggressive and infiltrative brain tumor 181. Monitoring CAR T cell activity after delivery is crucial because these challenges frequently reduce the effectiveness of CAR T cell treatment. Researchers can improve treatment approaches by using tracking techniques, which offer vital information on their movement, persistence, and interaction with the tumor. Multimodal imaging has been shown to be useful for tracking CAR T cells in GBM in a number of investigations. For instance, in mice models of GBM, researchers tracked the biodistribution and tumor buildup of CAR T cells tagged with Zirconium-89 using Positron Emission Tomography (PET) imaging. The study demonstrated that CAR T cells may effectively infiltrate tumor locations across the BBB, highlighting PET imaging's capacity to give quantitative 182.

Preclinical models have also made extensive use of bioluminescence imaging (BLI). To examine their growth and endurance within the tumor genetically modified CAR T cells to produce luciferase. BLI offered useful real-time insights into the dynamics of CAR T cell proliferation, although being restricted to small animal models because of its weak depth penetration 183. Similarly, CAR T cells that have been modified to produce fluorescent proteins like GFP or tagged with certain dyes have been used in preclinical settings to do fluorescence imaging. PET/MRI and PET/CT are examples of hybrid imaging methods that have significantly improved the precision of CAR T cell tracking. The combination of PET's sensitivity and MRI's anatomical resolution enhanced the visualization of CAR T cell distribution and tumor response, according to clinical research that used PET/MRI to track CAR T cells in a patient with recurrent GBM. These hybrid techniques offer a more thorough knowledge of CAR T cell function in the intricate tumor microenvironment of GBM, which makes them especially promising for clinical translation 184.

Future advancements in multimodal tracking are probably going to include synthetic biology and nanotechnology. Probes based on nanoparticles are being developed to deliver therapeutic payloads and improve imaging accuracy. By integrating synthetic genetic circuits into CAR T cells, researchers may directly observe tumor-targeting activity by using imaging signals that are triggered upon antigen contact 185. In order to provide prediction models of CAR T cell activity and therapeutic results, advances in AI and machine learning are also being investigated for the analysis of massive datasets produced by multimodal imaging. Multimodal *in vivo* tracking has enormous clinical promise. Key obstacles to the effectiveness of immunotherapy for GBM are addressed by these methods, which allow for accurate, real-time monitoring of CAR T cell migration, proliferation, and tumor interaction. For instance, a recent study showed that researchers may increase tumor invasion and durability by fine-tuning the design of CAR constructs by combining PET imaging and genetic analysis. In order to maximize CAR T cell therapy for GBM, this iterative strategy emphasizes how crucial it is to combine imaging and therapeutic development. Multimodal tracking is positioned to be crucial in developing CAR T cell immunotherapy for this debilitating illness as imaging technologies continue to advance 186.

Pfeifer *et al*. 2022 stated in his study that comprehensive spatiotemporal information is frequently lacking in CAR T cell studies in solid tumors, underscoring the necessity of sophisticated molecular tomography approaches to facilitate high-throughput preclinical surveillance. Furthermore, there is a crucial gap in understanding the intratumoral infiltration of therapeutic cells by integrating macro- and micro-level imaging data. In order to overcome these obstacles, they combined light-sheet fluorescence microscopy (LSFM), cyclic immunofluorescence staining (IF), and 3D computer tomography bioluminescence tomography (µCT/BLT) for a thorough examination. NSG mice with subcutaneous AsPC1 xenograft tumors were used in this work. Each set of seven mice received either EGFR CAR T cells (± IL-2) or control BDCA-2 CAR T cells (± IL-2). The luciferase CBR2opt and a particular CAR were co-expressed by the genetically modified CAR T cells. On days 1, 3, 5, and 7 after therapy, a subcutaneous injection of 25,000 IU of IL-2 was given to each mouse close to the xenograft tumor. Every three to four days, the dispersion of CAR T cells was tracked by 2D BLI and 3D µCT/BLT. On the sixth day, four mice's tumor samples were removed for cyclic IF labeling utilizing a panel of twenty-five antibodies. Furthermore, on days 6 and 13, tumors from mice that had received rhodamine lectin injections were removed, permeabilized, labeled for CD3, and photographed by LSFM. Results from 3D µCT/BLT showed that antigen recognition affects CAR T cell pharmacokinetics, with target-positive groups showing markedly higher tumor accumulation and delayed spleen accumulation in comparison to target-independent groups. By day six, LSFM verified deeper tumor penetration and higher CAR T cell infiltration in target-positive groups. Additionally, LSFM showed that CAR T cells tended to concentrate around blood arteries and at the tumor's periphery. As demonstrated by LSFM and cyclic IF, local IL-2 administration promoted the early-phase proliferation of CAR T cells; however, this was followed by long-term overstimulation, which ultimately negated the initial therapeutic effects. This study demonstrates the promise of 3D µCT/BLT as a reliable, non-isotope-based technique for examining the pharmacokinetics of CAR T cells and tracking whole-body cell treatment. The distribution and condition of therapeutic cells within tumor tissues may be thoroughly examined in both 2D and 3D thanks to the combination of LSFM with cyclic IF 187.

Kiru *et al.* 2022 reported in his study that with a two-year event-free survival rate of only 15% to 20%, metastatic osteosarcoma has a terrible prognosis, which highlights the urgent need for new potent treatments. A promising method for using the immune system to target cancers is CAR T-cell therapy. Nevertheless, despite its promise, CAR T cell clinical trials in solid tumors have encountered significant challenges and have not yet produced reliable results for a large patient group. The inability to use clinical imaging tools to track CAR T-cell accumulation within tumors is a major obstacle to the effectiveness of CAR T-cell treatment. They created a clinically useful technique to label CAR T cells with iron oxide nanoparticles in order to overcome this restriction. This allowed for the noninvasive viewing of the cells using magnetic particle imaging (MPI), photoacoustic imaging (PAT), and magnetic resonance imaging (MRI). They were able to sustain the viability, proliferation, and functionality of CAR T cells while achieving effective nanoparticle uptake by them using a specially made microfluidics device for mechanoporation. While animals treated with unlabeled CAR T cells displayed no discernible signal, multimodal imaging using MRI, PAT, and MPI was able to trace the migration of iron-labeled CAR T cells to osteosarcoma tumors and off-target areas in animal models. This study shows that CAR T cells can be effectively labeled with ferumoxytol, offering a viable method for tracking the behavior of CAR T cells in solid tumors 188.

**Figure 18:**
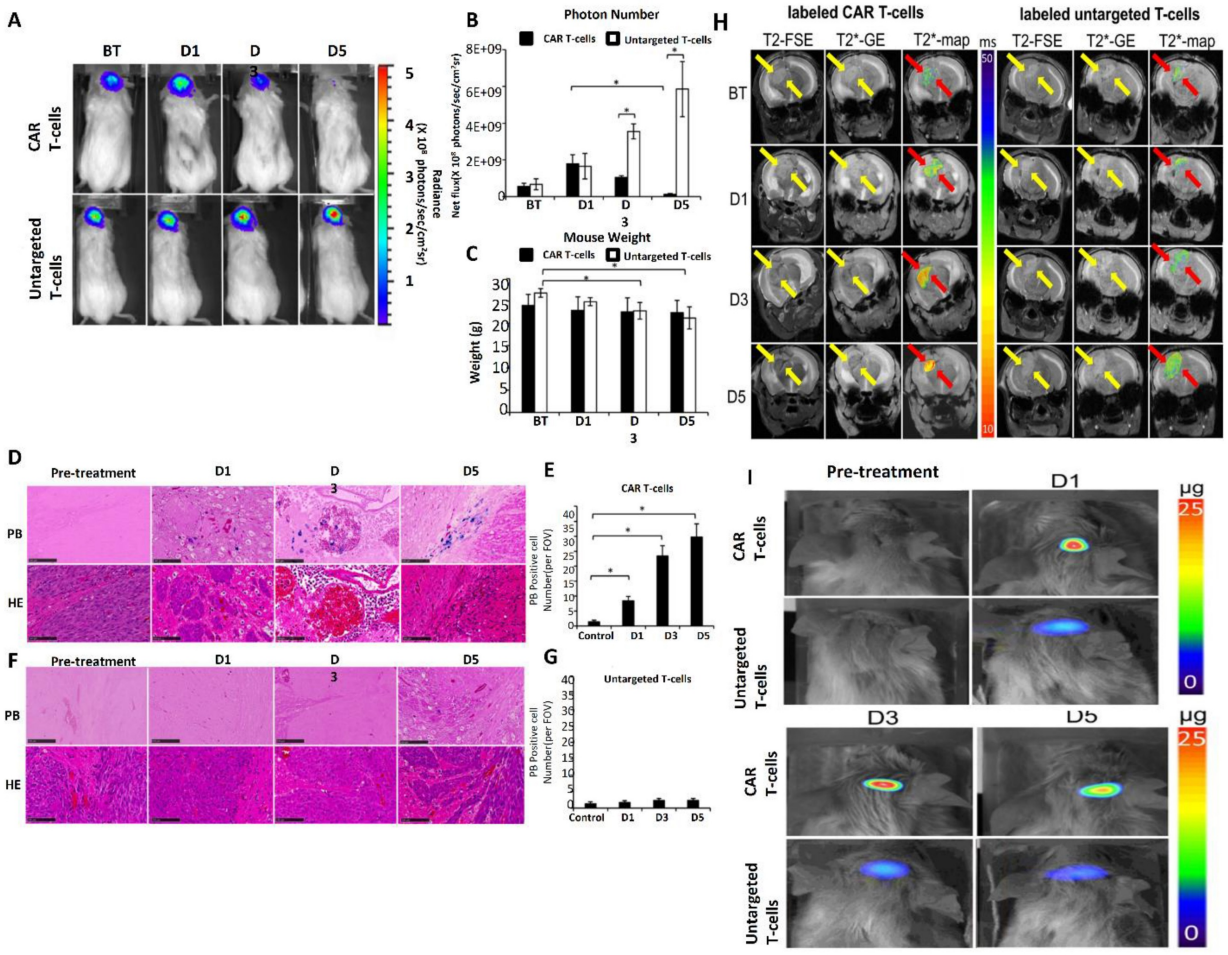
**(A)** Bioluminescence imaging (BLI) of a representative mouse treated with MegaPro-NP-labeled CAR T-cells is shown in the top row. **(B)** While tumor burden gradually rises after therapy with untargeted T-cells. **(C)** Mice treated with tagged untargeted T-cells gradually lost weight, whereas mice treated with labeled CAR T-cells showed steady body weight. **(D)** Analysis was done on tumor samples both before and after they were treated with MegaPro-NP-labeled CAR T-cells. **(E)** Prussian blue-positive cells increased over time after the infusion of tagged CAR T-cells, according to quantitative analysis. **(F)** By contrast, Prussian blue staining revealed no indication of iron-containing cells in tumor samples from mice treated with MegaPro-NP-labeled untargeted T-cells. **(G)** Quantitative data showed that there were few Prussian blue-positive cells in these samples, and H&E staining showed tumor shape without any discernible alterations (size bars: 100 µm). **(H)** Before treatment (BT), a hyperintense U87-MG tumor is shown on T2-weighted FSE and T2*-MGE imaging. On the left, the tumor shows a decrease in signal on days 1 (D1), 3 (D3), and 5 (D5) after MegaPro-NP-labeled CAR T-cells were infused. (I) MPI pictures, but mice treated with MegaPro-NP-labeled untargeted T-cells exhibit a weak MPI signal. Reused under Creative Commons Attribution License (CC-BY) 189.

According to Wu *et al.* (2022) magnetic resonance imaging (MRI) has been used to track the accumulation of CAR T cells using iron oxide nanoparticles. However, ferumoxytol, the only nanoparticle authorized for clinical usage, has been linked to infrequent but serious allergic responses. A safer substitute is MegaPro nanoparticles (MegaPro-NP). This work used a mouse model of GBM to examine MegaPro-NP's potential for *in vivo* monitoring of CAR T-cells. MegaPro-NP was used to label tumor-specific CD70CAR (8R-70CAR) T-cells and non-tumor-targeted control cells. Inductively coupled plasma optical emission spectroscopy (ICP-OES), Prussian blue staining, and cell survival assays were then used to evaluate the cells. Both tagged untargeted T-cells and MegaPro-NP-labeled or unlabeled CAR T-cells were administered to 42 NRG mice that had U87-MG/eGFP-fLuc GBM xenografts. Histological, MPI, and serial MRI studies were conducted, and statistical analyses were conducted using the Mann-Whitney U test for pairwise comparisons and the Kruskal-Wallis test for group differences. Without observable variations in viability, activation, or exhaustion signs (p>0.05), the results showed that CAR T-cells labeled with MegaPro-NP had much greater iron absorption than unlabeled cells (p<0.01). After treatment with MegaPro-NP-labeled CAR T-cells, *in vivo* imaging revealed considerably shorter tumor T2* relaxation durations than untargeted controls (p<0.01). Both mice treated with tagged and unlabeled CAR T-cells showed comparable tumor growth suppression. These results suggest that MegaPro-NP is a good choice for *in vivo* CAR T-cell tracking. MegaPro-NP is expected to be used soon to track CAR T-cells in cancer immunotherapy trials, as it recently finished Phase II clinical trials as an MRI contrast agent **Figure 18** 189.

## Role of AI for CAR T therapy

By tackling intricate therapeutic constraints and boosting the possibility of CAR T cell immunotherapy, artificial intelligence (AI) is transforming the study of GBM. Tumor heterogeneity, an immunosuppressive microenvironment, and the BBB are some of the major obstacles that GBM, a very aggressive brain tumor, faces. Through data-driven insights, AI provides sophisticated tools to enhance CAR T cell therapy, allowing for more individualized care and better patient results. AI is essential for identifying tumor antigens unique to GBM. AI is able to anticipate tumor-specific targets by analyzing vast datasets of genomic, transcriptomic, and proteomic profiles using machine learning algorithms. This makes it possible to create CAR T cells with more specificity, which lowers the possibility of side effects and increases their effectiveness against GBM cells 190.

AI also plays a crucial role in improving the generation and engineering of CAR T cells. In the immunosuppressive GBM microenvironment, AI-driven prediction models aid in the creation of T cells that demonstrate strong tumor invasion and durability. To increase the therapeutic potential of CAR designs, these models direct modifications such as adding dual antigen recognition or cytokine secretion pathways. AI-powered diagnostic and imaging technologies are also essential for tracking the effectiveness of treatment. Precise tracking of CAR T cells in the brain is made possible by sophisticated imaging algorithms, which help with real-time evaluation of tumor regression and any toxicities associated with therapy. These understandings enable prompt modifications to treatment plans, guaranteeing long-term effectiveness and reducing side effects 191.

**Figure 19:**
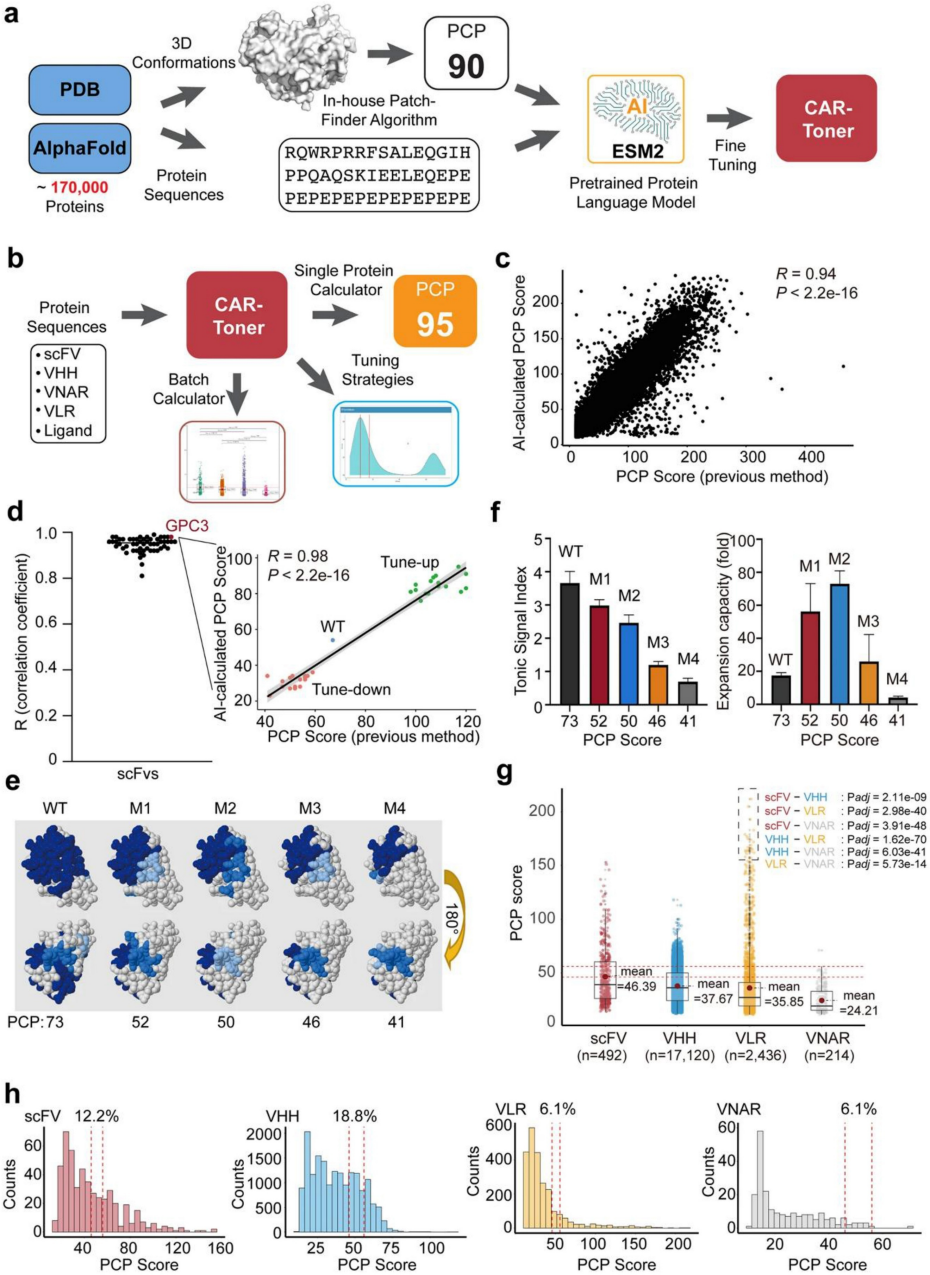
**(a)** A schematic illustration showing how CAR-Toner was developed. **(b)** The three major purposes of CAR-Toner are to compute the PCP of single or batch input sequences, optimize the PCP of the specified protein, and calculate the PCP of the CAR antigen-binding domain sequences. **(c)** Pearson correlation was used to evaluate the relationship between the AI-predicted PCP scores of protein sequences from the testing database using CAR-Toner and their PCP scores determined using an earlier technique. **(d)** CAR-Toner evaluation of the correlation coefficient for 54 scFvs. **(e)** Using the BindUP online server tool, see PCPs on the surface of CLL1 variant antibodies. The largest PCP is shown in dark blue, the second-largest PCP is shown in medium blue, and the third-largest PCP is shown in light blue. **(f)** Tonic signaling indices and CAR-T cell growth capabilities using mutant and wild-type (WT) CLL1 antibodies with different PCP scores. **(g)** PCP scores are calculated in batches using CAR-Toner for the scFv, VHH, VNAR, and VLR datasets. **(h)** The percentage of scFv, VHH, VNAR, and VLR datasets' antibody or antibody alternative sequences with PCP scores ranging from 46 to 56. Student's t-tests were used for statistical analysis of all comparisons. Reused under Creative Commons Attribution License (CC-BY) 192.

According to Qiu *et al*. (2024) CAR tonic signaling is essential for CAR-T cell activity because too much signaling causes CAR-T cell fatigue, while too little signaling results in poor persistence. Prior studies have demonstrated that CAR clustering is facilitated by positively charged patches (PCPs) on the surface of the CAR antigen-binding domain, which subsequently trigger tonic signaling. A bioinformatic method for determining the PCP score was previously devised in order to assess these PCPs, which are indicative of tonic signaling levels. Using the SWISS homology modeler, three-dimensional (3D) homology models of CAR single-chain variable fragments (scFvs) are created. Then, using the BindUP web server, residues within the top three largest surface patches of positively charged residues on the CAR scFv are identified and quantified. Nevertheless, this method has a number of shortcomings, such as reliance on outside servers, computation durations that take days for each sequence, inability to batch process, and lack of PCP score optimization tools. This research aims to overcome these constraints by creating an AI-based tool that can calculate and optimize PCP scores more effectively, removing current bottlenecks **Figure 19** 192.

## Challenges and Future Directions

Overcoming the highly immunosuppressive TME and BBB is one of the major obstacles to using CAR T-cell therapy to treat GBM 193. When CAR T cells arrive at the tumor site, they face a variety of challenges, such as immune cells, tumor-derived soluble substances, inhibitory cytokines, and metabolic and physical barriers. Within the GBM microenvironment, soluble molecules such prostaglandin E2, IL-6, IL-10, and TGF-β inhibit T-cell proliferation and effector activities 194. Compared to tumor samples obtained before treatment, surgical specimens showed a significant elevation of these immune suppressive factors following EGFRvIII CAR T-cell infusion. Furthermore, up to 30% of GBM-infiltrating lymphocytes are regulatory T cells (Tregs), which further suppress T-cell responses 195. Significant Treg cells infiltration was also observed in post-infusion tumor samples, as shown by the expression of CD4, CD25, and Forkhead box protein 3 196. Beyond Treg cells, GBM is characterized by a high concentration of tumor-associated macrophages, microglia, and myeloid-derived suppressor cells (MDSCs), all of which aid in the growth of the tumor 197. Notably, M2-polarized macrophages stimulate GBM stem cells and increase mitogen-activated protein kinase (MAPK) signaling, which aids in immune suppression and tumor support. It is also unclear how adaptive immune resistance develops in GBM after receiving various immunotherapies such dendritic cell vaccines. Preclinical research, however, indicates that PD-L1 adaptive overexpression could be a factor in treatment resistance 198.

Intratumoral heterogeneity is a significant obstacle to the long-term effectiveness of CAR T-cell treatment and is a primary cause of therapy resistance in GBM 199. Targets like EGFRvIII and IL-13Rα2 exhibit variable expression across temporal and geographic dimensions, as well as within and within individuals. For example, significant regional diversity in EGFRvIII expression was seen in biopsies taken from various parts of a single tumor in a patient receiving EGFRvIII CAR T cell treatment. This variation points to either varying CAR T-cell effectiveness at various tumor locations or, more likely, geographically variable EGFRvIII baseline expression. The phase III ACT IV study of rindopepimut, a peptide vaccination that targets EGFRvIII, had similar results. Regardless of treatment group, more than 50% of patients in this study displayed EGFRvIII antigen reduction in posttreatment tissue, highlighting the temporal heterogeneity in antigen expression 200.

These results cast doubt on the long-term viability of CAR T-cell treatments that target a single antigen. Clinical effectiveness may be restricted by antigen escape mechanisms unless CAR T cells are able to cause "antigen/epitope spreading" or indirect tumor cell death. CAR T cells stimulate endogenous CD8^+^ T-cell responses against non-targeted antigens by destroying target tumor cells, releasing stimulatory cytokines, and exposing tumor antigens in an active immune milieu. This process is known as antigen spreading. EGFRvIII CAR T cells may facilitate antigen dissemination, according to preclinical data, although its applicability in human GBM is yet unknown. Combinatorial strategies focusing on many tumor-associated antigens will be required if antigen spreading is shown to be inadequate. To better address tumor heterogeneity, current approaches include trivalent and bispecific CAR T-cell designs.

## Conclusion

One potential tumor immunotherapy that is especially well-suited to the difficulties presented by GBM is CAR T cell treatment. These CAR-T cells have the ability to enter the CNS, precisely destroy tumor cells, and reduce collateral harm. Additionally, payloads that boost the immunogenicity of the GBM microenvironment may be delivered by next-generation CAR T cells. Clinical studies have not yet been conducted despite encouraging preclinical data. However, new tactics are being created in reaction to these failures, which are revealing the barriers to success. Combination therapy, generated antigens, Image-guided CAR-T therap, use of AI and CAR T cells with multivalent receptors are being used to combat antigen escape. By fostering a more favorable, pro-inflammatory milieu, adjuvants used in conjunction with other immunotherapies, chemotherapies, or mechanically ablative techniques may also enhance outcomes. We will be closer to a successful immunotherapy for GBM if we keep working to better understand how the tumor, tumor microenvironment, and host immune system interact with CAR T treatment.

## Figures and Tables

**Table 1:** Summary of CAR-T-cell clinical trials in GBM.

Ref.	Target Antigen	Conditions	NCT No.	Phase	Lessons Learned
49	IL13Rα2	WHO Stage 3 or 4 unifocal supratentorial GBM recurring or resistant	NCT00730613	N/A(pilot study)	Repeated dosage, no negative side effects from therapy, and temporary anti-glioma action are all made possible by intracranial administration of CAR-T cells via a reservoir/catheter system.
50	IL13Rα2	Recurring multifocal GBM	NCT02208362	Phase I	2016: Case Report: Intraventricular infusion of CAR-T cells causes CNS malignancies, specifically spinal tumors, to recede; there is no systemic damage and the effect lasts for 7.5 months. 2024: For recurring GBM, along intraventricular and intratumoral administration of CAR-T demonstrated a prolonged survival; 50% of patients had stable tumors or greater, with two limited actions, one complete action, and a second complete response following extra CAR-T phases off protocol (7.7 months vs. 10.2 months).
51	IL13Rα2	Growing or recurring WHO Grade 3 or 4 malignant GBM	NCT01082926	Phase I	Indications for regional tumor necrosis, no graft-versus-host disease, and no side effects associated with the device
52	EGFRvIII	Recurrent GBM	NCT02209376	Phase I	Absence of syndrome of cytokines release or therapy-related toxic effects, CAR-T cell migration to the tumor spot, and elimination of the EGFRvIII antigen
53	EGFRvIII	Recurrent GBM	NCT01454596	Phase I	No toxicities that restrict dosage till the maximum dose (≥1010) no unbiased answers found.
54	EGFR and EGFRvIII	Recurrent GBM	NCT05660369	Phase I/pilot	Targeting EGFR variant III and wild-type EGFR, CARv3-TEAM-E T cells demonstrated encouraging safety profiles with no serious side effects or dose-limiting toxic effects, as well as temporary tumor shrinkage.
55	B7-H3	Recurrent GBM	-	-	Case Report: Short-term anti-tumor response generated by intraventricular T cells
